# Spatial analysis of COVID-19 cases and deaths among nursing professionals *

**DOI:** 10.1590/1518-8345.7400.4587

**Published:** 2025-10-27

**Authors:** Michelle Salles da Silva Tenorio, Paula Rita Dias de Brito de Carvalho, Keli Marini dos Santos Magno, Alexandre Sousa da Silva

**Affiliations:** 1 Universidade Federal do Estado do Rio de Janeiro, Escola de Enfermagem Alfredo Pinto, Rio de Janeiro, RJ, Brazil; 2 Secretaria Municipal de Saúde, Hospital Municipal Miguel Couto, Rio de Janeiro, RJ, Brazil; 3 Secretaria Estadual de Saúde, SES/RJ, Rio de Janeiro, RJ, Brazil; 4 Universidade Federal do Estado do Rio de Janeiro, Departamento de Métodos Quantitativos, Rio de Janeiro, RJ, Brazil

**Keywords:** COVID-19, Nurse Practitioners, Mortality, Working Conditions, Spatial Analysis, Secondary Data Analysis

## Abstract

to map cases and deaths from COVID-19 in nursing professionals, estimating their incidence and fatality rates by region and federative units, and verify the existence of spatial patterns among the federative units.

ecological study based on the electronic portals *Observatório de Enfermagem* and *Enfermagem em Números*, with analysis using the R 4.3.1 software. The incidence and fatality rates were calculated, and choropleth maps were constructed by region and federative units. The Global Moran Index was used to verify spatial autocorrelation.

the study covered 64,451 cases of COVID-19, with a female predominance (85.2%) and a focus on nursing technicians (59.3%), with a higher percentage in the Southeast region (36.3%). Deaths were predominantly female (68%), with the North region standing out (27.9%). The fatality rate in these cases was highest in the North region (4.25%) and in the state of Amazonas (28.47%). The spatial analysis showed regional variations, with autocorrelation in the overall case fatality rate in 2020 and for nursing technicians, without significant autocorrelation for other categories.

the mapping of COVID-19 cases and deaths in nursing revealed regional disparities and variations in incidence and fatality rates, highlighting the need to discuss the quality of health services and the effectiveness of the government response.

## Highlights

**(1)** COVID-19 has revealed disparities in health and infrastructure in Brazil across the five regions. **(2)** Professionals are experiencing precariousness, overload and shortages, making them vulnerable. **(3)** Response to COVID-19 must be adapted to local realities. **(4)** Developed regions have had better outcomes than less developed regions. **(5)** Study highlights the urgency of addressing and investing in vulnerable regions.

## Introduction

The COVID-19 pandemic has highlighted the need to discuss the quality of services and working conditions to which healthcare professionals are subjected^(1-2)^. With the spread of the disease, healthcare professionals faced an atypical work reality, characterized by a lack of knowledge about the disease, work overload, a shortage of tests with underestimation of cases, low adherence to social distancing measures, as well as the spread of fake news about COVID-19^(3-4)^.

Among healthcare professionals, Nursing, with approximately 2.3 million people in Brazil, stood out for its essential role on the front lines of the fight against COVID-19, acting both in care and in care management^(5-6)^. Representing 56% of the healthcare team, these professionals, who are considered the backbone of the healthcare workforce, faced considerable vulnerability, due to the nature of the work that significantly impacted the physical and mental health of these professionals^(5)^. Despite its outstanding importance and the large number of nursing professionals, in Brazil there is an unequal distribution of these professionals among and within states. States such as São Paulo, Rio de Janeiro and Minas Gerais have the largest numbers of professionals and, even in these, more than 50% work in the Brazilian capital cities^(7)^.

Brazil was one of the countries most affected by the COVID-19 pandemic, registering until January 2024, more than 38 million confirmed cases and more than 708,000 deaths in the general population^(8)^, with the distribution of these cases and deaths being quite heterogeneous among Brazilian states^(9)^.

Deeply diverse and unequal, Brazil is made up of a people of diverse cultural and ethnic-racial origins, as well as different religious traditions, not only separated by geographical borders, but also by historical, social and economic diversity. These characteristics, in turn, influenced exposure to COVID-19 and the severity of the disease, exacerbating vulnerability to infection and the risk of dying from the disease.

Since the beginning of the pandemic, several studies on COVID-19 have been widely conducted. International studies have sought to identify the main risk factors associated with COVID-19^(10)^, as well as the geographic location and populations most affected^(11)^. As for national studies, in addition to the themes already presented, they also sought to understand the repercussions on the mental health of the population^(12)^, as well as the challenges faced by health professionals in combating COVID-19^(1,13)^. Also in the Brazilian context, there are studies on the spatial distribution of cases, deaths and risk factors related to COVID-19 in the general population^(14-22)^, the distribution of health resources^(23)^ and the availability of beds^(15-16)^.

Among health professionals, nurses had a significantly higher mortality rate than that of doctors in Italy, Brazil, Spain and France^(24)^. In Brazil, data on COVID-19 cases and deaths among healthcare professionals are not easily accessible and, as a result, there are gaps to be filled^(22,25-26)^.

In this sense, understanding the spatial distribution of COVID-19 cases and deaths among nursing professionals can help public managers formulate data-based public policies, as well as support preventive measures to mitigate the risks to these professionals^(27-29)^.

Thus, the present study aimed to map COVID-19 cases and deaths among nursing professionals, estimating their incidence and fatality rates by region and federative units, and also to verify the existence of spatial patterns among federative units.

## Method

### Study design

This is an ecological study, descriptive in nature and quantitative in approach, based on open access secondary data. This study was conducted based on the recommendations of the STROBE checklist (Strengthening the Reporting of Observational Studies in Epidemiology).

### Used instruments for information collection

Data on cases and deaths among nursing professionals come from the electronic portal *Observatório de Enfermagem* ^(30)^ and data on the number of professionals come from *Enfermagem em Números* ^(31)^, a COFEN (Portuguese acronym for Federal Nursing Council) website that provides updated information on the number of active professional registrations, categorized by federative units of residence. It is important to note that the *Enfermagem em Números* portal is dynamically updated.

### Population and selection criteria

The study data correspond to records of cases and deaths from COVID-19 among nursing professionals throughout Brazil. The total sample of cases corresponded to 64,451 professionals affected by COVID-19, of which 872 professionals died^(31)^. The variables considered in the study were: Year (2020, 2021 and 2022); Federative Unit; Region; Professional Category (Assistant, Technician, Nurse and Midwife); Evolution (Death: yes or no); Gender (male and female); Age group (20-30; 31-40; 41-50; 51-60; 61-70; 71-80). The total number of professionals for each of the professional categories was also considered (data obtained from the Nursing in Numbers portal).

### Study scope scenario

The study covered notifications from nursing professionals throughout Brazil, in its regions and federative units. Brazil has 203.1 million inhabitants and is subdivided into 5 macro-regions: Central-West, Northeast, North, Southeast and South, which, in addition to the geographic division, present socioeconomic and cultural variations^(32)^.

The Central-West region, with 1.6 million km² and 16.2 million inhabitants, stands out for its territorial extension and the Pantanal. The Northeast, with 54.6 million inhabitants and 1.5 million km², is marked by social contrasts, cultural diversity and beaches. The North region, with more than 3.8 million km² and 17.3 million inhabitants, including indigenous and riverside communities, is characterized by the Amazon Rainforest and biodiversity. The Southeast region, with 84.8 million inhabitants and 41.8% of the Brazilian population, is the most populous, accounting for almost 50% of the gross domestic product (GDP), and stands out for its metropolitan regions. Finally, the South region, with 576 thousand km² and 29.9 million inhabitants, stands out for its economic diversity, European influence and high development rates^(32-35)^.

Regarding the number of nursing professionals, there are 2,706,308 registered professionals in the country, according to data from the Nursing in Numbers electronic portal. It is observed that the largest contingent of nursing professionals is found in the Southeast region, corresponding to 1,292,073 registered professionals; on the other hand, the North (227,774 professionals) and Central-West (200,726 professionals) regions have the smallest number of registered professionals. The number of professionals registered in the Southeast region corresponds almost to the sum of all professionals in the other regions of the country.

As for the states, reinforcing the hegemony of the Southeast region in relation to its economy and workforce, São Paulo (692,410 professionals) and Rio de Janeiro (329,104 professionals) are responsible for the largest number of professionals in the labor market. The state of Rio de Janeiro corresponds to the number of registrations of regions such as the South and Central-West, while the state of São Paulo corresponds to the Northeast region and has higher professional registrations than the other regions.

As for the distribution of the Brazilian nursing workforce across the study’s scope, coverage remains below the international recommendation (40 nurses for every 10,000 inhabitants). Furthermore, there is a considerable disparity in the coverage of professionals among the regions of Brazil, regions such as the Southeast and the South of the country have the highest rates of nurses per inhabitant, in contrast, regions such as the North and the Northeast have significantly lower numbers, often below 10 nurses for every 10 thousand inhabitants^(36)^.

### Data collection

To carry out the study, data on COVID-19 cases and deaths in Brazilian nursing were collected in August 2022 through the Nursing Observatory platform, including notification data for the period from March 2020 to July 2022. The choice of this collection interval is justified by the time lapse of death notifications on the Nursing Observatory platform, whose last death record occurred in January 2022. As for the nursing population data, from Nursing in Numbers, they were also collected in August 2022, and corresponded to the population of professionals registered with their respective Regional Nursing Councils in July 2022.

### Data analysis

In the exploratory analysis, frequency distributions were considered and incidence and lethality rates were calculated. Incidence rates were calculated using the ratio between COVID-19 cases in nursing professionals and the total number of nursing professionals from the Nursing in Numbers portal, with a multiplication factor of 100,000 inhabitants. To calculate lethality rates, the ratio between COVID-19 deaths in nursing professionals and COVID-19 cases in nursing professionals was considered, with a multiplication factor of 100.

For spatial analysis, choropleth maps of COVID-19 cases, deaths, incidence, and lethality in the federative units (UFs) were constructed. The maps were constructed using the grid of the Brazilian map by state, made available by the IBGE in 2022. To verify the existence of spatial autocorrelation, the Global Moran Index was considered, a measure that evaluates the differences between the values observed in each state and the global average of the observed attribute. The results of these measures range from -1 to 1, so that values close to zero suggest the absence of spatial correlation, values close to 1 indicate direct correlation, and values close to -1 indicate inverse correlation. As a criterion for defining the presence of neighborhood, the contiguity of borders was considered, thus, states that share borders are considered neighbors.

To carry out the study, the collected data were processed and analyzed using R software version 4.3.1, through the graphical interface R Studio^®^ version 2023.06.1 Build 524.

### Ethical aspects

Since this is secondary data in the public domain, in accordance with the provisions of CNS Resolution No. 466/12, this study did not require submission to the Research Ethics Committee^(37)^. Ethical and legal standards were respected, ratifying the authors’ commitment to the veracity of the data collected and the results presented.

## Results

The total study sample included 64,451 records of cases of nursing professionals infected with COVID-19, with a predominance of females (85.2%), as observed in Table 1. In order to construct the maps, occurrences without informed professional category were disregarded (3831 occurrences; 6% of cases). Table 1 addresses the cases, deaths by COVID-19 in nursing professionals according to sex, category, age group, year of notification and region of occurrence.

**Table 1-:** Cases and deaths from COVID-19 in nursing professionals according to professional category, gender, age group, year of notification and region of occurrence. Brazil, 2020-2022

		Cases n (%)	Deaths n (%)
Professional category	Nursing assistant	5,143 (8.0)	118 (13.5)
	Nursing technician	38,250 (59.3)	499 (57.2)
	Nurse	17,187 (26.7)	255 (29.2)
	Obstetrician	40 (<0.1)	0 (0.0)
	Not informed	3,831 (5.9)	0 (0.0)
Gender	Female	54,941 (85.2)	593 (68.0)
	Male	9,510 (14.8)	279 (32.0)
Age group (years)	20-30	14,317 (22.2)	34 (3.9)
	31-40	26,568 (41.2)	168 (19.2)
	41-50	16,977 (26.3)	270 (31.0)
	51-60	5,631 (8.7)	244 (28.0)
	61-70	876 (1.4)	138 (15.8)
	71-80	82 (0.1)	18 (2.1)
Notification year	2020	45,737 (71.0)	468 (53.7)
	2021	13,736 (21.3)	403 (46.2)
	2022	4,978 (7.7)	1 (0.1)
Region of occurrence	Midwest	5,388 (8.4)	128 (14.7)
	Northeast	16,627 (25.8)	154 (17.7)
	North	5,716 (8.9)	243(27.9)
	Southeast	23,395 (36.3)	238 (27.3)
	South	13,325 (20.7)	109 (12.5)
Total		64,451 (100.0)	872 (100.0)

From the perspective of the classification of cases by professional category, the largest contingent of occurrences was in the Nursing Technician category (59.3%) of cases. The analysis of cases by age group highlighted that the groups of 31-40 years (41.2%) and 41-50 years (26.3%) were the most affected. There was a significant drop in the distribution of cases over the years, from 71.0% in 2020 to 7.7% in 2022 (up to July). The Southeast region concentrated the highest percentage of cases (36.3%), while the North (8.9%) and Central-West (8.4%) regions recorded the lowest percentages.

Regarding deaths among Nursing professionals, the number was 872 deaths, with a predominance of females (68.0%) and a larger contingent of Nursing technicians (57.2%). The 41-50 age group had the highest number of deaths (270 deaths; 31.0%), followed by the 51-60 age group (244 deaths; 28.0%). Most deaths occurred in 2020 (468 deaths; 53.7%), with a small reduction in 2021 (403 deaths; 46.2%) and only one record until July 2022. The North Region had the highest percentage of deaths (243 deaths; 27.9%), followed by the Southeast Region (238 deaths; 27.3%).

**Figure 1-:**
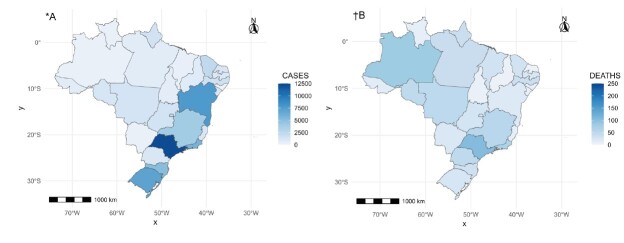
Map of COVID-19 cases and deaths in Brazilian states. Brazil, 2020-2022

Figure 1 shows the map of cases and deaths from COVID-19 by state. It is important to note that the state of São Paulo had a percentage of 19.1% with a total sample of 12,322 registered cases, almost equaling the entire South region of the country and exceeding the sum of the North and Central-West regions. The state with the second highest percentage was Bahia, with 12.0%, corresponding to 7,704 cases. Regarding deaths, São Paulo stands out with 105 (12% of the national total), followed by the state of Amazonas with 82 (9.4% of the national total) and Rio de Janeiro, which recorded 67 deaths of nursing professionals, corresponding to 7.7% of the national total.

*A = Cases due to COVID-19; ^†^B = Deaths due to COVID-19

Despite the large disparities in cases and deaths between the states, the Global Moran’s I Index did not show statistical significance (Cases: I = 0.087, p-value = 0.273; Deaths: I = 0.192, p-value = 0.058), indicating the absence of spatial autocorrelation.

Regarding incidence, it was observed that the South region had the highest value, corresponding to 3,978 cases/100,000 professionals, followed by the Central-West region with 2,684 cases/100,000 professionals, then the Northeast region with 2,555 cases/100,000 professionals, then the North region with 2,509 cases/100,000 professionals and finally the Southeast region with 1,811 cases/100,000 professionals.

**Figure 2-:**
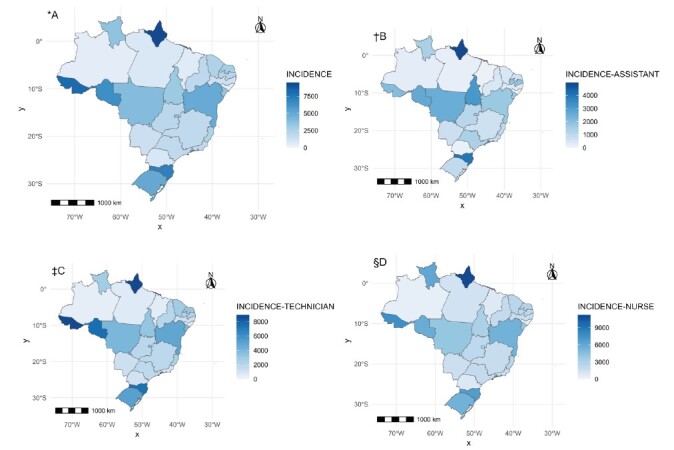
Map of incidence of COVID-19 in Nursing professionals in Brazilian states. Brazil, 2020-2022)

Figure 2 shows maps of the incidence rate by state. Figure 2 A shows the overall incidence, while the other images correspond to the incidence for assistants, technicians and nurses. It is observed that Amapá records the highest value in the three categories analyzed: nurses with 11,251 cases/100,000 professionals, nursing technicians with 8,941 cases/100,000 professionals, and nursing assistants with 4,973 cases/100,000 professionals. The state of Acre also stands out for technicians with 8,938 cases/100,000 professionals and nurses with 75.34 cases per 1,000 professionals, Rondônia for nursing technicians with 7,534 cases/100,000 professionals, and Santa Catarina for assistants with 3,957 cases/100,000 professionals and nursing technicians with 7,405 cases/100,000 professionals.

Regarding spatial autocorrelation, there is no autocorrelation for incidence rates (general: I = 0.016, p-value = 0.657; assistant: I = -0.113, p-value = 0.537; technician: I = 0.051, p-value = 0.476; nurse: I = -0.011, p-value = 0.820).

*A = Overall incidence of COVID-19; ^†^B = Incidence of COVID-19 in Nursing Assistants; ^‡^C = Incidence of COVID-19 in Nursing Technicians; ^§^D = Incidence of COVID-19 in Nurses

**Figure 3-:**
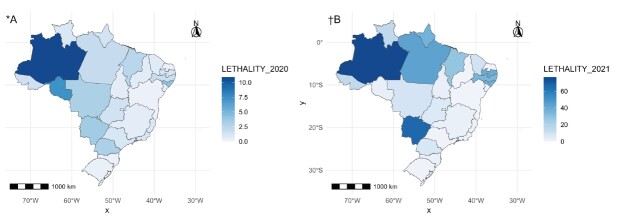
COVID-19 lethality in nursing according to the year of notification in the Brazilian states. Brazil, 2020-2021

Although more cases were observed in 2020, COVID-19 in 2021 was more lethal for nursing professionals (Table 1). Figure 3 shows the lethality by year of notification (2020 and 2021). Regarding spatial autocorrelation, it was observed for the year 2020: I = 0.241, p-value < 0.001, and in the year 2021: I = 0.014, p-value = 0.668. These results prove the presence of global spatial autocorrelation in 2020 and its absence in 2021. This indicates that neighboring states had similar lethality rates due to COVID-19. Furthermore, lethality rates were relatively low in 2020, ranging from 0.270 to 10.9 (Figure 3 A). Higher values were found in states in the North and Central-West regions, with emphasis on the state of Amazonas, while lower values were observed in the Southeast and Northeast states. With the advance of the pandemic in 2021, the spatial pattern was lost and there was an increase in the variability of lethality rates, starting from 0.0 for the states of Piauí and Rio Grande do Norte to 76.62 for Amazonas.

*A = COVID-19 lethality in 2020; ^†^B = COVID-19 lethality in 2021; No map was created for the year 2022, since there was only one death during the analysis

**Figure 4-:**
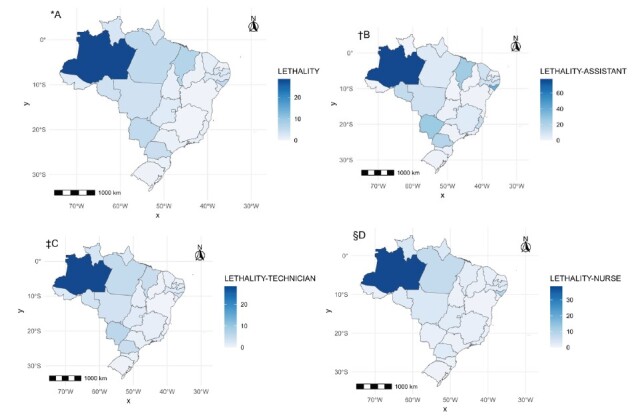
COVID-19 mortality in nursing and according to professional category in Brazilian states. Brazil, 2020-2022

Figure 4 presents maps of lethality among nursing professionals. Figure 4 A shows the grouped lethality for all categories, with emphasis on the state of Amazonas, which has the highest lethality, 28.74%, and the second highest for the state of Maranhão, with 6.7%. In all maps in Figure 4, the state of Amazonas stands out. For nursing assistants, the observed lethality was 77.78%; for nursing technicians, it was 38.57%; and for nurses, it was 28.04%.

*A = Overall lethality due to COVID-19; ^†^B = Lethality due to COVID-19 in Nursing Assistants; ^‡^C = Lethality due to COVID-19 in Nursing Technicians; ^§^D = Lethality due to COVID in nurses

Still regarding lethality among nursing professionals (Figure 4), the results for the Global Moran Index were: overall lethality: I = 0.084, p-value = 0.086; Assistant: I = -0.133, p-value = 0.349; Technician: I = 0.097, p-value = 0.037; Nurse: I = 0.037, p-value = 0.207. These results prove the absence of global spatial correlation for overall lethality, for nursing assistants and nurses, and the presence of spatial autocorrelation for technicians. Among nursing technicians, lethality stands out in the state of Amazonas, with 28.04%, while for the other states values lower than 6% were observed, with the lowest values recorded in coastal states, ranging from Rio Grande do Sul to Sergipe.

## Discussion

In order to deepen the understanding of the geographic distribution of the pandemic, this study mapped COVID-19 cases and deaths among nursing professionals, estimated their incidence and fatality rates by region and federal units, and analyzed the existence of spatial patterns among the different federal units.

Based on the analysis of the spatial distribution of COVID-19 cases and deaths, it is possible to identify areas of greater vulnerability, related to health infrastructure, access to resources, and working conditions. In addition, this analysis can provide support for the formulation of more effective public policies tailored to local needs. By mapping these inequalities, it is possible to direct actions that minimize the impacts of the pandemic and better prepare the health system for future crises.

The COVID-19 pandemic represented a major challenge for health professionals, especially for nursing professionals, who were essential in facing the crisis. Constantly exposed to contact with infected patients and involved in risky procedures, these professionals have become highly vulnerable to the disease^(38-39)^.

Nursing, considered a historically female profession (85.2%), has been showing a new trend of masculinization, with an increase in the male contingent^(40)^. According to data related to sex, although women represent twice the number of deaths in Brazilian Nursing in absolute numbers, men faced a higher risk of mortality from COVID-19, with a 45% lower probability of survival and a higher risk of death^(41)^. In addition, there is a higher number of hospitalizations for COVID-19 among the male population (54.4%), possibly due to negligence in relation to quarantine, lack of self-care, propensity for chronic diseases and, consequently, greater vulnerability to infection and disease severity^(42-45)^.

Although the association between advanced age and higher risk of mortality from COVID-19 is widely documented, other factors also play important roles^(38,43,46)^. Among nursing professionals, the age group of 31 to 40 years was the most affected by COVID-19 (41.2%), while 31% of deaths occurred among professionals aged 41 to 50 years, and 28% in the age group of 51 to 60 years. The fatality rates increase with age. In the states of Amazonas and Pará, the fatality rates were higher than 50% among nursing professionals in the older age groups. The fatality rate in the age group over 60 years, although the occurrence of deaths is lower, is worrying, as it reflects the loss of experienced professionals and those in leadership positions^(30,39,43)^.

In 2020, cases and deaths from COVID-19 were more numerous, but the fatality rates in 2021 exceeded those of the previous year in all regions of the country. States such as Amazonas, Mato Grosso do Sul, Pará and Amapá presented particularly high lethality rates, highlighting the vulnerability of the North region. The analysis over the three years of the pandemic, revealed a significant drop in cases and deaths among nursing professionals, reflecting the positive impact of vaccination that began in January 2021, with immunization demonstrating a protective effect among health professionals^(47)^. However, although the vaccinated variable was available on the Nursing Observatory platform, its inadequate completion prevented the use of the variable in this study.

The evolution of lethality patterns, as identified by the Global Moran Index, revealed a change in spatial autocorrelation between 2020 and 2021. In 2020, the presence of significant spatial autocorrelation indicated clusters of high lethality in neighboring states, suggesting a more uniform and coordinated response to the health emergency, with nearby regions sharing similar hospitalization and treatment conditions. However, in 2021, this spatial correlation dissipated, with case fatality varying more widely across states. This suggests that regional responses to COVID-19 have become more heterogeneous, highlighting the need for more adaptive public health strategies that are sensitive to local conditions.

Therefore, when establishing an analysis of the Brazilian scenario, the peculiarities of each of its macro-regions must be observed. During the pandemic, regional inequalities in Brazil were aggravated, and these results could have been mitigated by a government response adapted to local realities. It is important to consider that, in Brazil, more than 7 million inhabitants are located more than four hours from a city that is a hub for high-complexity care, with equipment, intensive care unit (ICU) beds available and specialized professionals. States such as Amazonas, Pará and Acre have more than 20% of their population requiring a commute of more than four hours for high-complexity care^(20,48)^.

Another relevant fact concerns the number of health plans clients. According to the National Supplementary Health Agency (ANS for the well-known Brazilian acronym), the Southeast region has the largest number of beneficiaries, followed by the South, Northeast and Central-West regions, with the North region having the lowest number of links to health plans^(49)^.

Furthermore, the availability of beds per inhabitant varies significantly between regions. The Southeast and Central-West regions had the largest supply of beds, with 11.50 and 10.07.

Therefore, when establishing an analysis of the Brazilian scenario, one must take into account the peculiarities of each of its macro-regions. During the pandemic, regional inequalities in Brazil were aggravated, and these results could have been mitigated by a government response adapted to local realities. It is important to consider that, in Brazil, more than 7 million inhabitants are located more than four hours from a city that is a hub for high-complexity care, with equipment, intensive care unit (ICU) beds available, and specialized professionals. States such as Amazonas, Pará, and Acre have more than 20% of their population requiring a travel time of more than four hours for high-complexity care^(20,48)^.

Another relevant fact concerns the number of beneficiaries of health plans. According to the National Supplementary Health Agency, the Southeast region has the largest number of beneficiaries, followed by the South, Northeast and Central-West regions, with the North region having the lowest number of health plan members^(49)^.

In addition, the availability of beds per inhabitant varies significantly between regions. The Southeast and Central-West regions had the largest supply of beds, with 11.50 and 10.07 beds per 100,000 inhabitants, respectively, while the North had the lowest coverage, with 7.46 beds per 100,000 inhabitants. The South region, in turn, stood out with the highest rate of beds exclusively for COVID-19 in the SUS, with 5.18 beds per 100,000 inhabitants^(50)^.

Amid the chaos of COVID-19, unequal access to public and supplementary health care may have significantly impacted the outcomes of the pandemic. Regions with greater penetration of public and private health care, such as the Southeast and South, had lower mortality rates and higher cure rates, in addition to a greater availability of hospital beds. In contrast, the North, a region with less availability, faced greater challenges in accessing health services, treatment and clinical support, resulting in more fatal outcomes^(49-50)^.

Brazil is a country of great diversity, with regions such as the North and Northeast presenting greater socioeconomic vulnerability. Although the Northeast has tourist attractions and some industrial presence, it faces significant challenges, such as low social indicators and a reduced GDP. The region has the second largest nursing workforce, but the coverage of professionals per inhabitant is lower than recommended, similar to that of the North^(31,36,51)^. In contrast, the Central-West, despite being less industrialized and having a low population density^(31,51)^, the Federal District had the highest coverage of nurses: 51.8 per 10,000 inhabitants in 2020, which may reflect on the quality of available health services^(36)^. A study on the spatial distribution of COVID-19 in Brazil revealed that regional inequalities influenced the spread and impacts of the disease, especially in areas with less availability of health professionals^(9)^.

The pandemic highlighted the fragility of access to health in the North region, which already faced challenges such as low human development indicators, long distances and precarious transportation infrastructure^(52)^. Despite the smaller number of professionals and low population density, the North region had the highest mortality rates. In comparison, the South, with high social indicators and approximately twice as many ICU beds per inhabitant, also had more mechanical ventilators and ICU doctors per capita. These resources are difficult to access and require years of specialized training to obtain the necessary qualifications^(20,51,53)^.

The overload of health services in Manaus, resulting from the concentration of highly complex care in the capital, resulted in the total occupancy of beds and the shortage of supplies, impacting COVID-19 statistics in other municipalities and compromising the response capacity of managers. With more than 50% of hospitalized patients originating from other locations, the demand for care went beyond municipal borders, forcing people to travel in search of services^(48,52)^.

Although the Northern region has a dispersed population and fewer urban centers, Manaus, with its high population density, faced a demand that exceeded the capacity of resources and professionals, even with the support of other states^(51)^. States in the Legal Amazon, such as Amapá, Amazonas and Roraima, recorded high rates of COVID-19, while health resources, such as ventilators and ICU beds, were allocated inefficiently, concentrating in the capitals and exacerbating inequality in access to health in the region^(23)^.

Despite having a relatively low distribution of cases, the state of Amazonas, the largest territorial extension in the country^(32)^, recorded the second highest number of deaths in the country, behind only São Paulo. This scenario reflects regional inequalities, such as the shortage of health professionals, the ineffectiveness of government actions and socioeconomic inequalities. The concentration of health services in the capitals, combined with existing precariousness, increases the risks for seriously ill patients, especially due to mobility challenges and the distribution of supplies. The lack of knowledge about health measures and low adherence to protocols further aggravate the vulnerability of the population, overloading health services^(52-53)^.

The spatial analysis of COVID-19 cases and deaths in Nursing was essential to identify geographic disparities and understand the complexity of the pandemic. It highlighted the need for a multifaceted approach in planning and formulating strategies, especially in vulnerable regions. This approach is essential to optimize public health responses, control the spread of the virus, and effectively reduce the impact of the disease^(53)^.

The results of this study make significant contributions to the advancement of scientific knowledge, especially through the usage of databases specific to the field of nursing, such as the Nursing Observatory and Nursing in Numbers, which are little explored in the academic literature. Using these sources, the study analyzed the geographic distribution of cases and deaths from COVID-19 among nursing professionals, incorporating data on incidence and lethality by state, identifying spatial patterns based on the Global Moran index and the representation of choropleth maps, allowing for a more in-depth analysis of the data. The findings of the study offer new perspectives for future research, focused on the regional needs of these professionals and the improvement of public health policies.

This study has some limitations, including the absence of extremely relevant variables in the database, such as socioeconomic data, race/ethnicity, and comorbidities, as well as inadequate completion of notification forms in variables such as age, professional category, and vaccinated professionals. It is also necessary to consider the risk of underreporting of cases and deaths, a major challenge in the analysis of COVID-19 data in Nursing. In addition, the study has limitations inherent to its ecological design, especially regarding the possibility of ecological fallacy or aggregation bias. This means that its conclusions are applicable to population aggregates and cannot be extrapolated to the individual level.

## Conclusion

The COVID-19 pandemic has highlighted the profound inequalities in the Brazilian health system, highlighting the precarious working conditions of nursing professionals and the unequal distribution of resources. Regions with poor infrastructure and greater socioeconomic vulnerability faced more severe challenges and more adverse outcomes, in contrast to more developed areas that presented more favorable outcomes, due to better social indicators and broader access to public and supplementary health care.

Although the analysis of the impacts of the pandemic showed a significant reduction in cases and deaths, associated with the advance of vaccination, the assessment of the effect on nursing professionals was compromised by the lack of data on vaccination coverage, due to inadequate completion of notification forms. This highlights the urgent need to improve data collection and analysis, in addition to strengthening evidence-based public policies, with the aim of ensuring more effective protection for both health professionals and the general population.

The study also highlights the importance of an approach adapted to local realities and an effective government response in formulating proposals and strategies aimed at promoting, protecting and assisting the health of frontline workers. To combat inequalities, it is necessary to reallocate resources to strengthen services and ensure the implementation of effective preventive measures. This study seeks to encourage reflection on how regional disparities can impact the pandemic in different ways, highlighting the urgency of a differentiated approach and greater government investment in more socioeconomically vulnerable areas.

## Data Availability

All data generated or analysed during this study are included in this published article.
