# Supplementary material for: Spatial analysis of COVID-19 cases and deaths among nursing professionals
Source: Rev Lat Am Enfermagem. 2025 Oct 27;33:e4587. doi: 10.1590/1518-8345.7400.4587 (PMC12562718; doi:10.1590/1518-8345.7400.4587)
Supplement: Translation

# Translation: Análise espacial dos casos e óbitos por COVID-19 entre profissionais de Enfermagem*

**DOI:** 10.1590/1518-8345.7400.4588

**Authors:** Michelle Salles da Silva Tenorio, Paula Rita Dias de Brito de Carvalho, Keli Marini dos Santos Magno, Alexandre Sousa da Silva

**Affiliations:** 1 Universidade Federal do Estado do Rio de Janeiro, Escola de Enfermagem Alfredo Pinto, Rio de Janeiro, RJ, Brasil; 2 Secretaria Municipal de Saúde, Hospital Municipal Miguel Couto, Rio de Janeiro, RJ, Brasil; 3 Secretaria Estadual de Saúde, SES/RJ, Rio de Janeiro, RJ, Brasil; 4 Universidade Federal do Estado do Rio de Janeiro, Departamento de Métodos Quantitativos, Rio de Janeiro, RJ, Brasil

**Keywords:** COVID-19, Profissionais de Enfermagem, Mortalidade, Condições de Trabalho, Análise Espacial, Análise de Dados Secundários

## Abstract

mapear casos e óbitos por COVID-19 em profissionais de Enfermagem, estimar suas taxas de incidência e letalidade por região e unidades federativas, e verificar a existência de padrões espaciais entre as unidades federativas.

estudo ecológico a partir dos portais eletrônicos Observatório de Enfermagem e Enfermagem em Números, com análise utilizando o *software* R 4.3.1. Foram calculadas as taxas de incidência e letalidade, e construídos mapas coropléticos por região e unidades federativas. O Índice de Moran Global foi empregado para verificar a autocorrelação espacial.

o estudo abrangeu 64.451 casos de COVID-19, com predominância feminina (85,2%) e destaque para técnicos de Enfermagem (59,3%), com maior percentual na região Sudeste (36,3%). Óbitos foram majoritariamente femininos (68%), destacando-se a região Norte (27,9%). A letalidade foi mais alta na região Norte (4,25%) e no Estado do Amazonas (28,47%). A análise espacial mostrou variações regionais, com autocorrelação na letalidade geral em 2020 e para técnicos de Enfermagem, sem autocorrelação significativa para outras categorias.

o mapeamento dos casos e óbitos por COVID-19 na Enfermagem revelou disparidades regionais e variações nas taxas de incidência e letalidade, destacando a necessidade de debater a qualidade dos serviços de saúde e a eficácia da resposta governamental.

## Destaques

**(1)** A COVID-19 revelou disparidades no Brasil, em saúde e infraestrutura, nas cinco regiões. **(2)** Profissionais sofrem precarização, sobrecarga e escassez, tornando-os vulneráveis. **(3)** Resposta à COVID-19 deve adaptar-se às realidades locais. **(4)** Regiões desenvolvidas tiveram melhores desfechos do que regiões menos desenvolvidas. **(5)** Estudo destaca urgência de abordagem e investimento em regiões vulneráveis.

## Introdução

A pandemia de COVID-19 colocou em evidência a necessidade de debater a qualidade dos serviços e as condições de trabalho a que estão submetidos os profissionais de saúde^(1-2)^. Com a disseminação da doença, os profissionais desse setor enfrentaram uma realidade laboral atípica, caracterizada pela falta de conhecimento sobre a doença, sobrecarga de trabalho, escassez de testes, com subestimação de casos, baixa adesão às medidas de distanciamento social, além da massiva propagação de *fake news* sobre a COVID-19^(3-4)^.

Dentre os profissionais de saúde, a Enfermagem, com cerca de 2,3 milhões de profissionais atuantes no Brasil, destacou-se por seu papel essencial na linha de frente do enfrentamento da COVID-19, atuando tanto na assistência quanto no gerenciamento do cuidado^(5-6)^. Representando 56% da equipe de saúde, esses profissionais, considerados a espinha dorsal da força de trabalho em saúde, enfrentaram considerável vulnerabilidade, devido à natureza do trabalho que impactou significativamente a saúde física e mental desses profissionais^(5)^. Apesar de sua destacada importância e do grande quantitativo de profissionais de Enfermagem, no Brasil observa-se uma distribuição desigual desses profissionais entre os estados, nas capitais e no interior. Estados como São Paulo, Rio de Janeiro e Minas Gerais apresentam os maiores quantitativos de profissionais e, mesmo nesses, mais de 50% atuam nas capitais^(7)^.

O Brasil foi um dos países mais afetados pela pandemia de COVID-19, registrando até janeiro de 2024 mais de 38 milhões de casos confirmados e mais de 708 mil mortes na população em geral^(8)^, com distribuição dos casos e mortes bastante heterogênea entre os estados brasileiros^(9)^.

Profundamente diverso e desigual, o Brasil é constituído por um povo de origens culturais e étnico-raciais diversas, além de diferentes tradições religiosas, não apenas separados por fronteiras geográficas, mas também pela diversidade histórica, social e econômica. Essas características, por sua vez, influenciaram a exposição à COVID-19 e a gravidade da doença, exacerbando a vulnerabilidade à infecção e ao risco de morrer pela doença.

Desde o início da pandemia, diversas pesquisas sobre a COVID-19 foram amplamente conduzidas. Estudos internacionais buscaram identificar os principais fatores de risco associados à COVID-19^(10)^, tanto quanto a localização geográfica e as populações mais afetadas^(11)^. Quanto aos estudos nacionais, além das temáticas já apresentadas, buscou-se compreender as repercussões na saúde mental da população^(12)^, assim como os desafios enfrentados pelos profissionais de saúde no combate à COVID-19^(1,13)^. Ainda no contexto brasileiro, há estudos sobre a distribuição espacial de casos, óbitos e fatores de risco relacionados à COVID-19 na população em geral^(14-22)^, a distribuição de recursos de saúde^(23)^ e a disponibilidade de leitos^(15-16)^.

Entre os profissionais de saúde, os enfermeiros apresentaram taxa de mortalidade significativamente maior do que a dos médicos na Itália, Brasil, Espanha e França^(24)^. No Brasil, os dados sobre casos e óbitos de COVID-19 em profissionais de saúde não são de fácil acesso e, por consequência, há lacunas a serem preenchidas^(22,25-26)^.

Nesse sentido, compreender a distribuição espacial de casos e óbitos de COVID-19 entre profissionais de Enfermagem poderá auxiliar os gestores públicos na formulação de políticas públicas fundamentadas em dados, bem como fundamentar medidas preventivas para mitigar os riscos desses profissionais^(27-29)^.

Assim, o presente estudo teve como objetivos: mapear casos e óbitos por COVID-19 em profissionais de Enfermagem, estimar suas taxas de incidência e letalidade por região, e unidades federativas, e verificar a existência de padrões espaciais entre as unidades federativas.

## Método

### Delineamento do estudo

Trata-se de um estudo ecológico, de caráter descritivo e abordagem quantitativa, a partir de dados secundários de acesso aberto. Este estudo foi conduzido com base nas recomendações do *checklist* STROBE (*Strengthening the Reporting of Observational Studies in Epidemiology*).

### Instrumentos utilizados para coleta das informações

Os dados sobre casos e óbitos em profissionais de Enfermagem são provenientes do portal eletrônico Observatório de Enfermagem^(30)^ e os dados sobre o quantitativo de profissionais são provenientes do Enfermagem em Números^(31)^, página eletrônica do Conselho Federal de Enfermagem (COFEN), que disponibiliza informações atualizadas acerca do quantitativo de registros profissionais ativos, categorizados por unidades federativas de residência. É importante salientar que o portal Enfermagem em Números é dinamicamente atualizado.

### População e critérios de seleção

Os dados do estudo correspondem aos registros de casos e óbitos por COVID-19 de profissionais de Enfermagem em todo o Brasil. A amostra total de casos correspondeu a 64.451 profissionais acometidos por COVID-19, dentre os quais 872 profissionais foram a óbito^(31)^. As variáveis consideradas no estudo foram: Ano (2020, 2021 e 2022); Unidade Federativa; Região; Categoria Profissional (Auxiliar, Técnico, Enfermeiro e Obstetriz); Evolução (Óbito: sim ou não); Sexo (masculino e feminino); Faixa etária (20-30; 31-40; 41-50; 51-60; 61-70; 71-80). Também foi considerado o total de profissionais para cada uma das categorias profissionais (dados obtidos no portal Enfermagem em Números).

### Cenário de abrangência do estudo

O estudo abrangeu as notificações de profissionais de Enfermagem de todo o território brasileiro, em suas regiões e unidades federativas. O Brasil apresenta 203,1 milhões de habitantes e é subdividido em 5 macrorregiões: Centro-Oeste, Nordeste, Norte, Sudeste e Sul, que, além da divisão geográfica, apresentam variações socioeconômicas e culturais^(32)^.

A região Centro-Oeste, com 1,6 milhões de km² e 16,2 milhões de habitantes, destaca- se pela extensão territorial e pelo Pantanal. O Nordeste, com 54,6 milhões de habitantes e 1,5 milhões de km², é marcado por contrastes sociais, diversidade cultural e praias. Já a região Norte, com mais de 3,8 milhões de km² e 17,3 milhões de habitantes, incluindo comunidades indígenas e ribeirinhas, sendo caracterizada pela Floresta Amazônica e biodiversidade. A região Sudeste, com 84,8 milhões de habitantes e 41,8% da população brasileira, é a mais populosa, concentrando quase 50% do produto interno bruto (PIB), destacando-se por suas regiões metropolitanas. Por fim, a região Sul, com 576 mil km² e 29,9 milhões de habitantes, se destaca pela diversidade econômica, influência europeia e altos índices de desenvolvimento^(32-35)^.

Com relação ao quantitativo de profissionais de Enfermagem, no país, há o registro de 2.706.308 profissionais, segundo dados do portal eletrônico Enfermagem em Números. É observado que o grande contingente da Enfermagem se encontra na região Sudeste, correspondendo a 1.292.073 profissionais registrados; por outro lado, as regiões Norte (227.774 profissionais) e Centro-Oeste (200.726 profissionais) apresentam o menor quantitativo de profissionais registrados. O quantitativo de profissionais registrados na região Sudeste corresponde quase ao somatório de todos os profissionais das demais regiões do país.

Quanto aos estados, reforçando a hegemonia da região Sudeste em relação à sua força de trabalho, São Paulo (692.410 profissionais) e Rio de Janeiro (329.104 profissionais) são responsáveis pelo maior quantitativo de profissionais no mercado de trabalho. O estado do Rio de Janeiro corresponde ao quantitativo de registros de regiões como Sul e Centro-Oeste, enquanto o estado de São Paulo corresponde à região Nordeste e tem registros profissionais superiores às demais regiões.

Quanto à distribuição da força de trabalho da Enfermagem brasileira pelo cenário de abrangência do estudo, a cobertura permanece aquém da recomendação internacional (40 enfermeiros a cada 10 mil habitantes). Além disso, há uma disparidade considerável na cobertura de profissionais entre as regiões do Brasil, regiões como Sudeste e o Sul do país possuem as maiores taxas de enfermeiros por habitantes, em contrapartida, regiões como o Norte e o Nordeste apresentam números significativamente mais baixos, frequentemente abaixo de 10 enfermeiros a cada 10 mil habitantes^(36)^.

### Coleta dos dados

Para a realização do estudo, foram coletados dados acerca dos casos e óbitos por COVID-19 na Enfermagem brasileira, em agosto de 2022, por meio da plataforma do Observatório de Enfermagem, compreendendo os dados de notificação referentes ao período de março de 2020 a julho de 2022. A escolha desse intervalo de coleta justifica-se pelo lapso temporal das notificações de óbitos na plataforma do Observatório de Enfermagem, cujo último registro de óbito ocorreu em janeiro de 2022. Quanto aos dados populacionais da Enfermagem, provenientes do Enfermagem em Números, também foram coletados, em agosto de 2022, e correspondiam à população de profissionais inscritos em seus respectivos Conselhos Regionais de Enfermagem no mês de julho de 2022.

### Análise dos dados

Na análise exploratória foram consideradas distribuições de frequência e calculadas taxas de incidência e letalidade. No cálculo das taxas de incidência, utilizou-se a razão entre os casos de COVID-19 em profissionais de Enfermagem e o número total de profissionais de Enfermagem, a partir do portal Enfermagem em Números, com fator de multiplicação por 100.000 habitantes. Para o cálculo das taxas de letalidade, considerou-se a razão entre os óbitos por COVID-19 em profissionais de Enfermagem e os casos de COVID-19 em profissionais de Enfermagem com fator de multiplicação por 100.

Para a análise espacial foram construídos mapas coropléticos dos casos, óbitos, incidência e letalidade por COVID-19 nas unidades federativas (UFs). A construção dos mapas utilizou a malha do mapa do Brasil por UF, disponibilizado pelo IBGE em 2022. Para verificar a existência de autocorrelação espacial, foi considerado o Índice de Moran Global, uma medida que avalia as diferenças entre os valores observados em cada UF e a média global do atributo observado. Os resultados dessas medidas variam de -1 a 1, de forma que valores próximos de zero sugerem ausência de correlação espacial, valores próximos de 1 indicam correlação direta e valores próximos de -1 correlação inversa. Como critério para definir a presença de vizinhança, considerou-se a contiguidade de fronteiras, assim, UFs que compartilham fronteira são consideradas vizinhas.

Para realização do estudo, os dados coletados foram processados e analisados utilizando o *software* R versão 4.3.1, por meio da interface gráfica *R Studio* ^®^ versão 2023.06.1 *Build* 524.

### Aspectos éticos

Por se tratar de dados secundários de domínio público, em conformidade com o que está disposto na Resolução CNS nº 466/12, este estudo não necessitou de submissão ao Comitê de Ética em Pesquisa^(37)^. Foram respeitadas as normas ético-legais, ratificando o compromisso dos autores quanto à veracidade dos dados coletados e resultados apresentados.

## Resultados

A amostra total do estudo abrangeu 64.451 registros de casos de profissionais de Enfermagem infectados com COVID-19, com predomínio do sexo feminino (85,2%), conforme observado na Tabela 1. Para a construção dos mapas, foram desconsideradas as ocorrências sem categoria profissional informada (3.831 ocorrências; 6% dos casos). A Tabela 1 aborda os casos, óbitos por COVID-19 em profissionais de Enfermagem segundo sexo, categoria, faixa etária, ano de notificação e região de ocorrência.

Sob a ótica da classificação de casos por categoria profissional, o maior contingente de ocorrências foi da categoria Técnico de Enfermagem (59,3%) dos casos. A análise dos casos por faixa etária destacou que os grupos de 31-40 anos (41,2%) e 41-50 anos (26,3%) foram os mais afetados. Houve uma queda significativa na distribuição de casos ao longo dos anos, de 71,0% em 2020 para 7,7% em 2022 (até julho). A região Sudeste concentrou o maior percentual de casos (36,3%), enquanto as regiões Norte (8,9%) e Centro-Oeste (8,4%) registraram os menores percentuais.

**Tabela 1-:** Casos e óbitos por COVID-19 em profissionais de Enfermagem segundo categoria profissional, sexo, faixa etária, ano de notificação e região de ocorrência. Brasil, 2020-2022

		**Casos n (%)**	**Óbitos n (%)**
Categoria profissional	Auxiliar de Enfermagem	5.143 (8,0)	118 (13,5)
	Técnico de Enfermagem	38.250 (59,3)	499 (57,2)
	Enfermeiro	17.187 (26,7)	255 (29,2)
	Obstetriz	40 (<0,1)	0 (0,0)
	Não informado	3831 (5,9)	0 (0,0)
Sexo	Feminino	54.941 (85,2)	593 (68,0)
	Masculino	9.510 (14,8)	279 (32,0)
Faixa etária (anos)	20-30	14.317 (22,2)	34 (3,9)
	31-40	26.568 (41,2)	168 (19,2)
	41-50	16.977 (26,3)	270 (31,0)
	51-60	5.631 (8,7)	244 (28,0)
	61-70	876 (1,4)	138 (15,8)
	71-80	82 (0,1)	18 (2,1)
Ano de notificação	2020	45.737 (71,0)	468 (53,7)
	2021	13.736 (21,3)	403 (46,2)
	2022	4.978 (7,7)	1 (0,1)
Região de ocorrência	Centro-Oeste	5.388 (8,4)	128 (14,7)
	Nordeste	16.627 (25,8)	154 (17,7)
	Norte	5.716 (8,9)	243(27,9)
	Sudeste	23.395 (36,3)	238 (27,3)
	Sul	13.325 (20,7)	109 (12,5)
Total		64.451 (100,0)	872 (100,0)

Com relação aos óbitos entre os profissionais de Enfermagem, o quantitativo foi 872 óbitos, com predominância do sexo feminino (68,0%) e maior contingente de técnicos de Enfermagem (57,2%). O intervalo de 41-50 anos teve o maior número de óbitos (270 óbitos; 31,0%), seguido por 51-60 anos (244 óbitos; 28,0%). A maioria dos óbitos ocorreu em 2020 (468 óbitos; 53,7%), com uma pequena redução em 2021 (403 óbitos; 46,2%) e apenas um registro até julho de 2022. A Região Norte teve o maior percentual de óbitos (243 óbitos; 27,9%), seguida pela Região Sudeste (238 óbitos; 27,3%).

Na Figura 1 apresenta-se o mapa de casos e óbitos por COVID-19 por UF. É importante salientar que o estado de São Paulo apresentou um percentual de 19,1% com amostra total de 12.322 casos registrados, quase equiparando a toda região Sul do país e superando o somatório das regiões Norte e Centro-Oeste. O estado com o segundo maior percentual foi a Bahia, apresentando 12,0%, correspondendo a 7.704 casos. Com relação ao óbito, São Paulo se destaca com 105 (12% do total nacional), seguido pelo estado do Amazonas com 82 (9,4% do total nacional) e pelo Rio de Janeiro, que registrou 67 mortes de profissionais de enfermagem, o que corresponde a 7,7% do total nacional.

**Figura 1-:** Mapa de casos e óbitos por COVID-19 nos estados brasileiros. Brasil, 2020-2022

*A = Casos por COVID-19; ^†^B = Óbitos por COVID-19

Apesar das grandes disparidades de casos e óbitos entre as UFs, o Índice I de Moran Global não apresentou significância estatística (Casos: I = 0,087, p-valor = 0,273; Óbitos: I = 0,192, p-valor = 0,058), apontando para a ausência de autocorrelação espacial.

Com relação à incidência, observou-se que a região Sul apresenta o maior valor correspondendo a 3.978 casos/100.000 profissionais, seguida pela região Centro-Oeste com 2.684 casos/100.000 profissionais, posteriormente região Nordeste com 2.555 casos/100.000 profissionais, depois a região Norte com 2.509 casos/100.000 profissionais e por fim, pela região Sudeste com 1.811 casos/100.000 profissionais.

Na Figura 2 são apresentados mapas da taxa de incidência por UF. A Figura 2 A apresenta a incidência geral, enquanto as demais imagens correspondem à incidência para os auxiliares, técnicos e enfermeiros. Observa-se que o Amapá registra o maior valor nas três categorias analisadas: enfermeiros com 11.251 casos/100.000 profissionais, técnicos de Enfermagem com 8.941 casos/100.000 profissionais, e auxiliares de Enfermagem com 4.973 casos/100.000 profissionais. Destacam-se também o estado do Acre para técnicos com 8.938 casos/100.000 profissionais e enfermeiros com 75,34 casos por 1000 profissionais, Rondônia para técnicos de Enfermagem com 7.534 casos/100.000 profissionais, e Santa Catarina para auxiliares com 3.957 casos/100.000 profissionais e técnicos de Enfermagem com 7.405 casos/100.000 profissionais. Com relação à autocorrelação espacial, comprova-se ausência de autocorrelação para as taxas de incidência (geral: I = 0,016, p-valor =0,657; auxiliar: I = -0,113, p-valor = 0,537; técnico: I = 0,051, p-valor = 0,476; enfermeiro: I = -0,011, p-valor = 0,820).

**Figura 2-:** Mapa de incidência de COVID-19 em profissionais de Enfermagem nos estados brasileiros. Brasil, 2020-2022

*A = Incidência geral por COVID-19; ^†^B = Incidência por COVID-19 em Auxiliares de Enfermagem; ^‡^C = Incidência por COVID-19 em Técnicos de Enfermagem; ^§^D = Incidência por COVID-19 em Enfermeiros

Apesar de terem sido observados mais casos no ano de 2020, a COVID-19 em 2021 foi mais letal para profissionais de Enfermagem (Tabela 1). A Figura 3 apresenta a letalidade por ano de notificação (2020 e 2021). Com relação à autocorrelação espacial, observou-se para o ano de 2020: I = 0,241, p-valor < 0,001, e no ano de 2021: I = 0,014, p-valor = 0,668. Estes resultados comprovam a presença de autocorrelação espacial global no ano de 2020 e ausência em 2021. Isso indica que as UFs vizinhas apresentaram letalidade por COVID-19 semelhantes, além disso, as taxas de letalidade foram relativamente baixas em 2020, variando de 0,270 a 10,9 (Figura 3 A). Valores mais altos foram encontrados em UFs das regiões Norte e Centro-Oeste, com destaque para o estado do Amazonas, enquanto valores mais baixos foram observados nos estados do Sudeste e Nordeste. Com o avanço da pandemia em 2021, o padrão espacial se perdeu e houve aumento na variabilidade das taxas de letalidade, partindo de 0,0 para os estados do Piauí e Rio Grande do Norte a 76,62 para o Amazonas.

**Figura 3-:** Letalidade por COVID-19 da Enfermagem segundo ano de notificação nos estados brasileiros. Brasil, 2020-2021

*A = Letalidade por COVID-19 em 2020; ^†^B = Letalidade por COVID-19 em 2021; Não foi confeccionado mapa para o ano de 2022, uma vez que houve apenas um óbito durante a análise

Na Figura 4, são apresentados mapas da letalidade em profissionais de Enfermagem. A Figura 4 A mostra a letalidade agrupada para todas as categorias, com destaque para o estado do Amazonas, que apresenta a maior letalidade, de 28,74%, e o segundo maior para o estado do Maranhão, com 6,7%. Em todos os mapas da Figura 4, o estado do Amazonas se destaca. Para auxiliares de Enfermagem, a letalidade observada foi de 77,78%; para técnicos de Enfermagem, foi de 38,57%; e para enfermeiros, foi de 28,04%.

**Figura 4-:** Letalidade por COVID-19 na Enfermagem e segundo a categoria profissional nos estados brasileiros. Brasil, 2020-2022

*A = Letalidade geral por COVID-19; ^†^B = Letalidade por COVID-19 em Auxiliares de Enfermagem; ^‡^C = Letalidade por COVID-19 em Técnicos de Enfermagem; ^§^D = Letalidade por COVID-19 em Enfermeiros

Ainda com relação à letalidade entre os profissionais de Enfermagem (Figura 4), os resultados para o Índice de Moran Global foram: letalidade geral: I = 0,084, p-valor = 0,086; Auxiliar: I = -0,133, p-valor = 0,349; Técnico: I = 0,097, p-valor = 0,037; Enfermeiro: I = 0,037, p-valor = 0,207. Esses resultados comprovam a ausência de correlação espacial global para a letalidade geral, para os auxiliares de Enfermagem e para os enfermeiros, e a presença de autocorrelação espacial para os técnicos. Entre os técnicos de Enfermagem, a letalidade se destaca no estado do Amazonas, com 28,04%, enquanto para os demais estados foram observados valores menores que 6%, com os valores mais baixos registrados em estados da costa, variando do Rio Grande do Sul a Sergipe.

## Discussão

Com o intuito de aprofundar a compreensão sobre a distribuição geográfica da pandemia, o presente estudo mapeou os casos e óbitos por COVID-19 em profissionais de Enfermagem, estimou suas taxas de incidência e letalidade por região e unidades federativas, bem como analisou a existência de padrões espaciais entre as diferentes unidades da federação.

A partir da análise da distribuição espacial de casos e óbitos por COVID-19, é possível identificar áreas de maior vulnerabilidade, relacionadas à infraestrutura de saúde, ao acesso a recursos e às condições de trabalho. Além disso, essa análise pode fornecer subsídios para a formulação de políticas públicas mais eficazes e ajustadas às necessidades locais. Ao mapear essas desigualdades, é possível direcionar ações que minimizem os impactos da pandemia e preparem melhor o sistema de saúde para crises futuras.

A pandemia de COVID-19 representou um grande desafio para os profissionais de saúde, especialmente para os de Enfermagem, que foram essenciais no enfrentamento da crise. Constantemente expostos ao contato com pacientes infectados e envolvidos em procedimentos de risco, esses profissionais tornaram-se altamente vulneráveis à doença^(38-39)^.

A Enfermagem, considerada uma profissão historicamente feminina (85,2%), vem apresentando uma nova tendência, com aumento do contingente masculino^(40)^. Segundo os dados relacionados ao sexo, embora as mulheres representem o dobro dos óbitos na Enfermagem brasileira em números absolutos, os homens enfrentaram um risco maior de mortalidade por COVID-19, com uma probabilidade de sobrevivência 45% menor e maior risco de óbito^(41)^. Além disso, há um número maior de hospitalizações por COVID-19 entre o público masculino (54,4%), possivelmente devido à negligência em relação à quarentena, déficit no autocuidado, propensão a doenças crônicas e, consequentemente, maior vulnerabilidade à infecção e à gravidade da doença^(42-45)^.

Embora a associação entre idade avançada e maior risco de mortalidade por COVID-19 seja amplamente documentada, outros fatores também desempenham papéis importantes^(38,43,46)^. Entre os profissionais de Enfermagem, a faixa etária de 31 a 40 anos foi a mais acometida por COVID-19 (41,2%), enquanto 31% dos óbitos ocorreram entre profissionais de 41 a 50 anos, e 28% na faixa de 51 a 60 anos. As taxas de letalidade aumentam com a idade. Nos estados do Amazonas e Pará, as taxas de letalidade foram superiores a 50% entre os profissionais de Enfermagem nas faixas etárias mais avançadas. A letalidade na faixa etária acima de 60 anos, embora a ocorrência de óbitos seja menor, é preocupante, pois reflete a perda de profissionais experientes e em posições de liderança^(30,39,43)^.

Em 2020, os casos e óbitos por COVID-19 foram mais numerosos, mas as taxas de letalidade em 2021 superaram as do ano anterior em todas as regiões do país. Estados como Amazonas, Mato Grosso do Sul, Pará e Amapá apresentaram letalidades particularmente elevadas, destacando a vulnerabilidade da região Norte. A análise ao longo dos três anos da pandemia, revelou uma queda significativa nos casos e óbitos entre os profissionais de Enfermagem, refletindo o impacto positivo da vacinação iniciada em janeiro de 2021, com a imunização demonstrando efeito protetor entre os profissionais de saúde^(47)^. Contudo, embora a variável “vacinados” estivesse disponível na plataforma Observatório da Enfermagem, seu preenchimento inadequado impediu a sua utilização neste estudo.

A evolução dos padrões de letalidade, conforme identificado pelo Índice de Moran Global, revelou uma mudança na autocorrelação espacial entre 2020 e 2021. Em 2020, a presença de autocorrelação espacial significativa indicava clusters de alta letalidade em estados vizinhos, sugerindo uma resposta mais uniforme e coordenada à emergência sanitária, com regiões próximas compartilhando condições semelhantes de hospitalização e tratamento. No entanto, em 2021, essa correlação espacial se dissipou, com a letalidade variando mais amplamente entre as UFs. Isso sugere que as respostas regionais ao COVID-19 se tornaram mais heterogêneas, destacando a necessidade de estratégias de saúde pública mais adaptativas e sensíveis às condições locais.

Assim, ao estabelecer uma análise do cenário brasileiro, deve-se observar as peculiaridades de cada uma de suas macrorregiões. Durante a pandemia, as desigualdades regionais no Brasil foram agravadas, e esses resultados poderiam ter sido mitigados por uma resposta governamental adaptada às realidades locais. É importante considerar que, no Brasil, mais de 7 milhões de habitantes estão localizados a mais de quatro horas de um município polo de atendimento de alta complexidade, com equipamentos, leitos de unidade de terapia intensiva (UTI) disponíveis e profissionais especializados. Estados como Amazonas, Pará e Acre apresentam mais de 20% de sua população necessitando de deslocamento superior a quatro horas para atendimento de alta complexidade^(20,48)^.

Outro dado relevante diz respeito ao número de beneficiários de planos de saúde. Segundo a Agência Nacional de Saúde Suplementar (ANS), a região Sudeste é a que apresenta o maior número de beneficiários, seguida pelas regiões Sul, Nordeste e Centro- Oeste, sendo a região Norte a que apresenta o menor número de vínculos com planos de saúde^(49)^.

Além disso, a disponibilidade de leitos por habitante varia significativamente entre as regiões. O Sudeste e o Centro-Oeste apresentaram a maior oferta de leitos, com 11,50 e 10,07 leitos por 100.000 habitantes, respectivamente, enquanto o Norte teve a menor cobertura, com 7,46 leitos por 100.000 habitantes. A região Sul, por sua vez, destacou-se com a maior taxa de leitos exclusivos para COVID-19 no SUS, com 5,18 leitos por 100.000 habitantes^(50)^.

Em meio ao caos da COVID-19, o acesso desigual à saúde pública e suplementar pode ter impactado significativamente os desfechos da pandemia. Regiões com maior presença da saúde pública e privada, como Sudeste e Sul, apresentaram menores taxas de mortalidade e maiores percentuais de cura, além de uma maior disponibilidade de leitos de internação. Em contrapartida, o Norte, região com menor disponibilidade, enfrentou maiores desafios no acesso aos serviços de saúde, tratamento e suporte clínico, resultando em desfechos mais fatais^(49-50)^.

O Brasil é um país de grande diversidade, com regiões como o Norte e o Nordeste apresentando maior vulnerabilidade socioeconômica. Embora o Nordeste tenha atrativos turísticos e alguma presença industrial, enfrenta desafios significativos, como baixos indicadores sociais e um PIB reduzido. A região possui a segunda maior força de trabalho na Enfermagem, mas a cobertura de profissionais por habitante é inferior ao preconizado, similar à do Norte^(31,36,51)^. Em contraste, no Centro-Oeste, apesar de ser menos industrializado e ter baixa densidade populacional^(31,51)^, o Distrito Federal apresentou a maior cobertura de enfermeiros: 51,8 por 10 mil habitantes, em 2020, o que pode refletir na qualidade dos serviços de saúde disponíveis^(36)^. Um estudo sobre a distribuição espacial da COVID-19 no Brasil revelou que as desigualdades regionais influenciaram a disseminação e os impactos da doença, especialmente em áreas com menor disponibilidade de profissionais de saúde^(9)^.

A pandemia evidenciou a fragilidade do acesso à saúde na região Norte, que já enfrentava desafios como baixos indicadores de desenvolvimento humano, longas distâncias e infraestrutura de transporte precária^(52)^. Apesar da menor quantidade de profissionais e da baixa densidade populacional, a região Norte apresentou as maiores taxas de letalidade. Em comparação, o Sul, com altos indicadores sociais e aproximadamente o dobro de leitos de UTI por habitante, contava também com mais ventiladores mecânicos e médicos de UTI *per capita*. Esses recursos são de difícil acesso e exigem anos de treinamento especializado para a qualificação necessária^(49,51,53)^.

A sobrecarga dos serviços de saúde em Manaus, decorrente da concentração da assistência de alta complexidade na capital, resultou na total ocupação dos leitos e na escassez de insumos, impactando as estatísticas de COVID-19 em outros municípios e comprometendo a capacidade de resposta dos gestores. Com mais de 50% dos pacientes internados originários de outras localidades, a demanda por atendimento ultrapassou as fronteiras municipais, forçando deslocamentos em busca de serviços^(48,52)^.

Apesar de a região Norte apresentar uma população dispersa e menos centros urbanos, Manaus, com sua alta densidade populacional, enfrentou uma demanda que superou a capacidade de recursos e profissionais, mesmo com o apoio de outros estados^(51)^. Estados da Amazônia Legal, como Amapá, Amazonas e Roraima, registraram altas taxas de COVID-19, enquanto os recursos de saúde, como ventiladores e leitos de UTI, foram alocados de maneira ineficiente, concentrando-se nas capitais e exacerbando a desigualdade no acesso à saúde na região^(23)^.

O Amazonas, estado de maior extensão territorial do país^(32)^, apesar de apresentar uma distribuição relativamente baixa de casos, registrou o segundo maior número de óbitos no país, ficando atrás apenas de São Paulo. Esse cenário reflete as desigualdades regionais, como a escassez de profissionais de saúde, a ineficácia das ações governamentais e as desigualdades socioeconômicas. A concentração dos serviços de saúde nas capitais, somada às precariedades existentes, aumenta os riscos para pacientes graves, especialmente devido aos desafios de mobilidade e à distribuição de insumos. A falta de conhecimento sobre medidas sanitárias e a baixa adesão aos protocolos agravam ainda mais a vulnerabilidade da população, sobrecarregando os serviços de saúde^(52-53)^.

A análise espacial dos casos e óbitos de COVID-19 na Enfermagem foi essencial para identificar disparidades geográficas e compreender a complexidade da pandemia. Ela evidenciou a necessidade de uma abordagem multifacetada no planejamento e na formulação de estratégias, especialmente em regiões vulneráveis. Essa abordagem é fundamental para otimizar as respostas em saúde pública, controlar a disseminação do vírus e reduzir o impacto da doença de maneira eficaz^(53)^.

Os resultados do presente estudo trazem contribuições significativas para o avanço do conhecimento científico, especialmente pelo uso de bases de dados específicas da área da Enfermagem, como o Observatório da Enfermagem e Enfermagem em Números, que são pouco exploradas na literatura acadêmica. A partir dessas fontes, o estudo analisou a distribuição geográfica de casos e óbitos por COVID-19 entre profissionais de Enfermagem, incorporando dados sobre incidência e letalidade por UF, identificando padrões espaciais a partir do índice de Moran Global e da representação de mapas coropléticos, permitindo uma análise mais aprofundada dos dados. Os achados do estudo oferecem novas perspectivas para investigações futuras, voltadas às necessidades regionais desses profissionais e ao aprimoramento das políticas públicas de saúde.

Este estudo apresenta algumas limitações, dentre elas, a ausência de variáveis de extrema relevância no banco de dados, como dados socioeconômicos, raça/etnia e comorbidades, bem como o preenchimento inadequado das fichas de notificação em variáveis como idade, categoria profissional e profissionais vacinados, sendo necessário também considerar o risco de subnotificação de casos e óbitos, um grande desafio na análise dos dados de COVID-19 na Enfermagem. Além disso, o estudo apresenta limitações inerentes ao seu delineamento ecológico, especialmente no que se refere à possibilidade de falácia ecológica ou viés de agregação. Isso significa que suas conclusões são aplicáveis a agregados populacionais e não podem ser extrapoladas para o nível individual.

## Conclusão

A pandemia de COVID-19 evidenciou as profundas desigualdades no sistema de saúde brasileiro, destacando a precarização das condições de trabalho dos profissionais de Enfermagem e a distribuição desigual de recursos. Regiões com infraestrutura precária e maior vulnerabilidade socioeconômica enfrentaram desafios mais severos e resultados mais adversos, em contraste com áreas mais desenvolvidas que apresentaram desfechos mais favoráveis, devido a melhores indicadores sociais e acesso mais amplo à saúde pública e suplementar.

Embora a análise dos impactos da pandemia tenha mostrado uma significativa redução de casos e óbitos, associada ao avanço da vacinação, a avaliação do efeito em profissionais de Enfermagem foi comprometida pela falta de dados sobre a cobertura vacinal, devido ao preenchimento inadequado das fichas de notificação. Isso ressalta a necessidade urgente de aprimorar a coleta e análise de dados, além de fortalecer políticas públicas baseadas em evidências, com o objetivo de garantir uma proteção mais eficaz tanto para os profissionais de saúde quanto para a população em geral.

O estudo também sublinha a importância de uma abordagem adaptada às realidades locais e de uma resposta governamental eficaz na formulação de propostas e estratégias voltadas para a promoção, proteção e assistência à saúde dos profissionais na linha de frente. Para combater as desigualdades, é necessário realocar recursos para fortalecer os serviços e garantir a implementação de medidas preventivas eficazes. Esse estudo busca fomentar a reflexão sobre como as disparidades regionais podem impactar a pandemia de maneira diversa, evidenciando a urgência de uma abordagem diferenciada e de um maior investimento governamental em áreas socioeconomicamente mais vulneráveis.

## Data Availability

Todos os dados gerados ou analisados durante este estudo estão incluídos neste artigo publicado.

# Translation: Análisis espacial de los casos y fallecimientos por COVID-19 entre profesionales de Enfermería*

**DOI:** 10.1590/1518-8345.7400.4586

**Authors:** Michelle Salles da Silva Tenorio, Paula Rita Dias de Brito de Carvalho, Keli Marini dos Santos Magno, Alexandre Sousa da Silva

**Affiliations:** 1 Universidade Federal do Estado do Rio de Janeiro, Escola de Enfermagem Alfredo Pinto, Rio de Janeiro, RJ, Brasil; 2 Secretaria Municipal de Saúde, Hospital Municipal Miguel Couto, Rio de Janeiro, RJ, Brasil; 3 Secretaria Estadual de Saúde, SES/RJ, Rio de Janeiro, RJ, Brasil; 4 Universidade Federal do Estado do Rio de Janeiro, Departamento de Métodos Quantitativos, Rio de Janeiro, RJ, Brasil

**Keywords:** COVID-19, Enfermeras Practicantes, Mortalidad, Condiciones de Trabajo, Análisis Espacial, Análisis de Datos Secundarios

## Abstract

mapear casos y fallecimientos por COVID-19 en profesionales de Enfermería, estimar sus tasas de incidencia y letalidad por región y unidades federativas, y verificar la existencia de patrones espaciales entre las unidades federativas.

estudio ecológico a partir de los portales electrónicos Observatorio de Enfermería y Enfermería en Números, con análisis utilizando el *software* R 4.3.1. Se calcularon las tasas de incidencia y letalidad, y se construyeron mapas coropléticos por región y unidades federativas. Se empleó el Índice de Moran Global para verificar la autocorrelación espacial.

el estudio abarcó 64.451 casos de COVID-19, con predominio femenino (85,2%) y destaque para técnicos de Enfermería (59,3%), con mayor porcentaje en la región Sudeste (36,3%). Los fallecimientos fueron mayoritariamente femeninos (68%), destacándose la región Norte (27,9%). La letalidad fue más alta en la región Norte (4,25%) y en el Estado de Amazonas (28,47%). El análisis espacial mostró variaciones regionales, con autocorrelación en la letalidad general en 2020 y para técnicos de Enfermería, sin autocorrelación significativa para otras categorías.

el mapeo de los casos y fallecimientos por COVID-19 en Enfermería reveló disparidades regionales y variaciones en las tasas de incidencia y letalidad, destacando la necesidad de debatir la calidad de los servicios de salud y la eficacia de la respuesta gubernamental.

## Destacados

**(1)** La COVID-19 reveló disparidades en Brasil, en salud e infraestructura, en las cinco regiones. **(2)** Los profesionales sufren precarización, sobrecarga y escasez, volviéndolos vulnerables. **(3)** La respuesta a la COVID-19 debe adaptarse a las realidades locales. **(4)** Las regiones desarrolladas tuvieron mejores resultados que las regiones menos desarrolladas. **(5)** El estudio destaca la urgencia de un enfoque y de inversión en regiones vulnerables.

## Introducción

La pandemia de COVID-19 puso de manifiesto la necesidad de debatir la calidad de los servicios y las condiciones de trabajo a las que están sometidos los profesionales de la salud^(1-2)^. Con la difusión de la enfermedad, los profesionales de la salud enfrentaron una realidad laboral atípica, caracterizada por la falta de conocimiento sobre la enfermedad, sobrecarga de trabajo, escasez de pruebas con subestimación de casos, baja adhesión a las medidas de distanciamiento social, así como la propagación de *fake news* sobre la COVID-19^(3-4)^.

Entre los profesionales de la salud, la Enfermería, con cerca de 2,3 millones en Brasil, se destacó por su papel esencial en la primera línea del enfrentamiento de la COVID-19, actuando tanto en la asistencia como en la gestión del cuidado^(5-6)^. Representando el 56% del equipo de salud, estos profesionales, que son considerados la columna vertebral de la fuerza laboral en salud, enfrentaron una considerable vulnerabilidad, debido a la naturaleza del trabajo que impactó significativamente la salud física y mental de estos profesionales^(5)^. A pesar de su destacada importancia y del gran número de profesionales de Enfermería, en Brasil se observa una distribución desigual de estos profesionales entre los estados y, también, dentro de ellos. Estados como São Paulo, Rio de Janeiro y Minas Gerais presentan los mayores números de profesionales y, incluso en estos, más del 50% actúan en las capitales^(7)^.

Brasil fue uno de los países más afectados por la pandemia de COVID-19, registrando hasta enero de 2024, más de 38 millones de casos confirmados y más de 708 mil muertes en la población en general^(8)^, con una distribución de estos casos y muertes bastante heterogénea entre los estados brasileños^(9)^.

Profundamente diverso y desigual, Brasil está constituido por un pueblo de orígenes culturales y étnico-raciales diversos, además de diferentes tradiciones religiosas, no solo separados por fronteras geográficas, sino también por diversidad histórica, social y económica. Estas características, a su vez, influenciaron la exposición a la COVID-19 y la gravedad de la enfermedad, exacerbando la vulnerabilidad a la infección y el riesgo de morir a causa de la enfermedad.

Desde el inicio de la pandemia, se han realizado numerosas investigaciones sobre la COVID-19. Estudios internacionales han buscado identificar los principales factores de riesgo asociados a la COVID-19^(10)^, así como la localización geográfica y las poblaciones más afectadas^(11)^. En cuanto a los estudios nacionales, además de las temáticas ya presentadas, también se ha buscado comprender las repercusiones en la salud mental de la población^(12)^, así como los desafíos enfrentados por los profesionales de la salud en el combate contra la COVID-19^(1,13)^. Aún en el contexto brasileño, existen estudios sobre la distribución espacial de casos, muertes y factores de riesgo relacionados con la COVID-19 en la población en general^(14-22)^, la distribución de recursos de salud^(23)^ y la disponibilidad de camas^(15-16)^.

Entre los profesionales de la salud, los enfermeros presentaron una tasa de mortalidad significativamente mayor que la de los médicos en Italia, Brasil, España y Francia^(24)^. En Brasil, los datos sobre casos y muertes por COVID-19 en profesionales de la salud no son de fácil acceso y, en consecuencia, existen lagunas que deben ser llenadas^(22,25-26)^.

En este sentido, comprender la distribución espacial de casos y muertes por COVID-19 entre profesionales de Enfermería podrá ayudar a los gestores públicos en la formulación de políticas públicas fundamentadas en datos, así como fundamentar medidas preventivas para mitigar los riesgos de estos profesionales^(27-29)^.

Así, el presente estudio tuvo como objetivos: mapear casos y muertes por COVID-19 en profesionales de Enfermería, estimar sus tasas de incidencia y letalidad por región, y unidades federativas, y verificar la existencia de patrones espaciales entre las unidades federativas.

## Método

### Delineamiento del estudio

Se trata de un estudio ecológico, de carácter descriptivo y enfoque cuantitativo, a partir de datos secundarios de acceso abierto. Este estudio fue conducido con base en las recomendaciones del *checklist* STROBE (*Strengthening the Reporting of Observational Studies in Epidemiology*).

### Instrumentos utilizados para la recolección de información

Los datos sobre casos y muertes en profesionales de Enfermería son provenientes del portal electrónico Observatorio de Enfermería^(30)^ y los datos sobre el número de profesionales son provenientes de Enfermería en Números^(31)^, página electrónica del COFEN, que dispone de información actualizada acerca del número de registros profesionales activos, categorizados por unidades federativas de residencia. Es importante señalar que el portal Enfermería en Números se actualiza de manera dinámica.

### Población y criterios de selección

Los datos del estudio corresponden a los registros de casos y muertes por COVID-19 de profesionales de Enfermería en todo Brasil. La muestra total de casos correspondió a 64.451 profesionales afectados por COVID-19, de los cuales 872 profesionales fallecieron^(31)^. Las variables consideradas en el estudio fueron: Año (2020, 2021 y 2022); Unidad Federativa; Región; Categoría Profesional (Auxiliar, Técnico, Enfermero y Obstetra); Evolución (Fallecimiento: sí o no); Sexo (masculino y femenino); Rango de edad (20-30; 31-40; 41-50; 51-60; 61-70; 71-80). También se consideró el total de profesionales para cada una de las categorías profesionales (datos obtenidos en el portal Enfermería en Números).

### Escenario de alcance del estudio

El estudio abarcó las notificaciones de profesionales de Enfermería de todo el territorio brasileño en sus regiones y unidades federativas. Brasil presenta 203,1 millones de habitantes y se subdivide en 5 macrorregiones: Centro-Oeste, Nordeste, Norte, Sudeste y Sur, que, además de la división geográfica, presentan variaciones socioeconómicas y culturales^(32)^.

La región Centro-Oeste, con 1,6 millones de km² y 16,2 millones de habitantes, se destaca por su extensión territorial y por el Pantanal. El Nordeste, con 54,6 millones de habitantes y 1,5 millones de km², se caracteriza por contrastes sociales, diversidad cultural y playas. La región Norte, con más de 3,8 millones de km² y 17,3 millones de habitantes, incluyendo comunidades indígenas y ribereñas, se caracteriza por la Selva Amazónica y biodiversidad. La región Sudeste, con 84,8 millones de habitantes y el 41,8% de la población brasileña, es la más poblada, concentrando casi el 50% del producto interno bruto (PIB), destacándose por sus regiones metropolitanas. Por último, la región Sur, con 576 mil km² y 29,9 millones de habitantes, se destaca por la diversidad económica, la influencia europea y altos índices de desarrollo^(32-35)^.

En cuanto al número de profesionales de Enfermería, en el país hay un registro de 2.706.308 profesionales, según datos del portal electrónico Enfermería en Números. Se observa que el gran contingente de la Enfermería se encuentra en la región Sudeste, correspondiendo a 1.292.073 profesionales registrados; por otro lado, las regiones Norte (227.774 profesionales) y Centro-Oeste (200.726 profesionales) presentan el menor número de profesionales registrados. El número de profesionales registrados en la región Sudeste corresponde casi a la suma de todos los profesionales de las demás regiones del país.

En cuanto a los estados, reforzando la hegemonía de la región Sudeste con relación a su fuerza laboral, São Paulo (692.410 profesionales) y Rio de Janeiro (329.104 profesionales) son responsables del mayor número de profesionales en el mercado laboral. El estado de Rio de Janeiro corresponde al número de registros de regiones como el Sur y Centro-Oeste, mientras que el estado de São Paulo corresponde a la región Nordeste y cuenta con registros profesionales superiores a los de las demás regiones.

Con relación a la distribución de la fuerza laboral de la Enfermería brasileña en el ámbito del estudio, la cobertura se encuentra por debajo de la recomendación internacional (40 enfermeros cada 10 mil habitantes). Además, existe una disparidad considerable en la cobertura de profesionales entre las regiones de Brasil, ya que regiones como el Sudeste y el Sur del país poseen las mayores tasas de enfermeros por habitantes, en contraposición a regiones como el Norte y el Nordeste que presentan números significativamente más bajos, frecuentemente por debajo de 10 enfermeros cada 10 mil habitantes^(36)^.

### Recolección de datos

Para la realización del estudio, se recopilaron datos acerca de los casos y fallecimientos por COVID-19 en la Enfermería brasileña, en agosto de 2022, mediante la plataforma del Observatorio de Enfermería, abarcando los datos de notificación correspondientes al período de marzo de 2020 a julio de 2022. La elección de este intervalo de recolección se justifica por el lapso temporal de las notificaciones de fallecimientos en la plataforma del Observatorio de Enfermería, cuyo último registro de fallecimiento se realizó en enero de 2022. En cuanto a los datos poblacionales de la Enfermería, provenientes de Enfermería en Números, también se recopilaron en agosto de 2022, y correspondían a la población de profesionales inscritos en sus respectivos Consejos Regionales de Enfermería en el mes de julio de 2022.

### Análisis de los datos

En el análisis exploratorio se consideraron distribuciones de frecuencia y se calcularon tasas de incidencia y letalidad. En el cálculo de las tasas de incidencia se utilizó la razón entre los casos de COVID-19 en profesionales de Enfermería y el número total de profesionales de Enfermería a partir del portal Enfermería en Números, con un factor de multiplicación por 100.000 habitantes. Para el cálculo de las tasas de letalidad se consideró la razón entre los fallecimientos por COVID-19 en profesionales de Enfermería y los casos de COVID-19 en profesionales de Enfermería, con un factor de multiplicación por 100.

Para el análisis espacial se construyeron mapas coropléticos de los casos, fallecimientos, incidencia y letalidad por COVID-19 en las unidades federativas (UFs). La construcción de los mapas utilizó la malla del mapa de Brasil por UF, disponible por el IBGE en 2022. Para verificar la existencia de autocorrelación espacial, se consideró el Índice de Moran Global, una medida que evalúa las diferencias entre los valores observados en cada UF y la media global del atributo observado. Los resultados de estas medidas varían de -1 a 1, de manera que valores cercanos a cero sugieren ausencia de correlación espacial, valores cercanos a 1 indican correlación directa y valores cercanos a -1, correlación inversa. Como criterio para definir la presencia de vecindad, se consideró la contigüidad de fronteras, de modo que las UFs que comparten frontera son consideradas vecinas.

Para la realización del estudio, los datos recopilados fueron procesados y analizados utilizando el *software* R versión 4.3.1, a través de la interfaz gráfica *R Studio* ^®^ versión 2023.06.1 *Build* 524.

### Aspectos éticos

Al tratarse de datos secundarios de dominio público, conforme a lo dispuesto en la Resolución CNS nº 466/12, este estudio no requirió la presentación al Comité de Ética en Investigación^(37)^. Se respetaron las normas ético-legales, ratificando el compromiso de los autores en cuanto a la veracidad de los datos recopilados y los resultados presentados.

## Resultados

La muestra total del estudio abarcó 64.451 registros de casos de profesionales de Enfermería infectados con COVID-19, con predominio del sexo femenino (85,2%), según se observa en la Tabla 1. Para la construcción de los mapas se desestimaron las ocurrencias sin categoría profesional informada (3831 ocurrencias; 6% de los casos). La Tabla 1 abordó los casos, fallecimientos por COVID-19 en profesionales de Enfermería según sexo, categoría, rango etario, año de notificación y región de ocurrencia.

**Tabla 1-:** Casos y fallecimientos por COVID-19 en profesionales de Enfermería según categoría profesional, sexo, rango etario, año de notificación y región de ocurrencia. Brasil, 2020-2022

		**Casos n (%)**	**Fallecimientos n (%)**
Categoría profesional	Auxiliar	5.143 (8,0)	118 (13,5)
	Técnico	38.250 (59,3)	499 (57,2)
	Enfermero	17.187 (26,7)	255 (29,2)
	Obstetra	40 (<0,1)	0 (0,0)
	No informado	3831 (5,9)	0 (0,0)
Sexo	Femenino	54.941 (85,2)	593 (68,0)
	Masculino	9.510 (14,8)	279 (32,0)
Rango etario (años)	20-30	14.317 (22,2)	34 (3,9)
	31-40	26.568 (41,2)	168 (19,2)
	41-50	16.977 (26,3)	270 (31,0)
	51-60	5.631 (8,7)	244 (28,0)
	61-70	876 (1,4)	138 (15,8)
	71-80	82 (0,1)	18 (2,1)
Año de notificación	2020	45.737 (71,0)	468 (53,7)
	2021	13.736 (21,3)	403 (46,2)
	2022	4.978 (7,7)	1 (0,1)
Región de ocurrencia	Centro-Oeste	5.388 (8,4)	128 (14,7)
	Nordeste	16.627 (25,8)	154 (17,7)
	Norte	5.716 (8,9)	243(27,9)
	Sudeste	23.395 (36,3)	238 (27,3)
	Sur	13.325 (20,7)	109 (12,5)
Total		64.451 (100,0)	872 (100,0)

Desde la perspectiva de la clasificación de casos por categoría profesional, el mayor contingente de ocurrencias fue de la categoría Técnico de Enfermería (59,3%) de los casos. El análisis de los casos por rango etario destacó que los grupos de 31-40 años (41,2%) y 41-50 años (26,3%) fueron los más afectados. Hubo una caída significativa en la distribución de casos a lo largo de los años, de 71,0% en 2020 a 7,7% en 2022 (hasta julio). La región Sudeste concentró el mayor porcentaje de casos (36,3%), mientras que las regiones Norte (8,9%) y Centro-Oeste (8,4%) registraron los menores porcentajes.

En cuanto a los fallecimientos entre los profesionales de Enfermería, el total fue de 872 fallecimientos, con predominio del sexo femenino (68,0%) y mayor contingente de técnicos de Enfermería (57,2%). El intervalo de 41-50 años presentó el mayor número de fallecimientos (270 fallecimientos; 31,0%), seguido por el rango de 51-60 años (244 fallecimientos; 28,0%). La mayoría de los fallecimientos ocurrió en 2020 (468 fallecimientos; 53,7%), con una pequeña reducción en 2021 (403 fallecimientos; 46,2%) y solo un registro hasta julio de 2022. La Región Norte registró el mayor porcentaje de fallecimientos (243 fallecimientos; 27,9%), seguida por la Región Sudeste (238 fallecimientos; 27,3%).

En la Figura 1 se presenta el mapa de casos y fallecimientos por COVID-19 por UF. Es importante señalar que el estado de São Paulo presentó un porcentaje del 19,1% con un total de 12.322 casos registrados, casi equiparando a toda la región Sur del país y superando la suma de las regiones Norte y Centro-Oeste. El estado con el segundo mayor porcentaje fue Bahía, presentando el 12,0%, lo que corresponde a 7.704 casos. En cuanto a los fallecimientos, São Paulo se destaca con 105 (12% del total nacional), seguido por el estado de Amazonas con 82 (9,4% del total nacional) y por Rio de Janeiro, que registró 67 muertes de profesionales de enfermería, lo que corresponde al 7,7% del total nacional.

**Figura 1-:** Mapa de casos y fallecimientos por COVID-19 en los estados brasileños. Brasil, 2020-2022

*A = Casos por COVID-19; ^†^B = Fallecimientos por COVID-19

A pesar de las grandes disparidades de casos y fallecimientos entre las UFs, el Índice I de Moran Global no presentó significancia estadística (Casos: I = 0,087, p-valor = 0,273; Fallecimientos: I = 0,192, p-valor = 0,058), señalando la ausencia de autocorrelación espacial.

En cuanto a la incidencia, se observó que la región Sur presenta el mayor valor, correspondiendo a 3.978 casos/100.000 profesionales, seguida por la región Centro-Oeste con 2.684 casos/100.000 profesionales, luego la región Nordeste con 2.555 casos/100.000 profesionales, después la región Norte con 2.509 casos/100.000 profesionales y, por último, la región Sudeste con 1.811 casos/100.000 profesionales.

En la Figura 2 se presentan mapas de la tasa de incidencia por UF. La Figura 2 A presenta la incidencia general, mientras que las demás imágenes corresponden a la incidencia para auxiliares, técnicos y enfermeros. Se observa que Amapá registra el mayor valor en las tres categorías analizadas: enfermeros con 11.251 casos/100.000 profesionales, técnicos de Enfermería con 8.941 casos/100.000 profesionales, y auxiliares de Enfermería con 4.973 casos/100.000 profesionales. También se destacan el estado de Acre para técnicos con 8.938 casos/100.000 profesionales y enfermeros con 75,34 casos por 1000 profesionales, Rondônia para técnicos de Enfermería con 7.534 casos/100.000 profesionales, y Santa Catarina para auxiliares con 3.957 casos/100.000 profesionales y técnicos de Enfermería con 7.405 casos/100.000 profesionales.

En cuanto a la autocorrelación espacial, se comprueba la ausencia de autocorrelación para las tasas de incidencia (general: I = 0,016, p-valor = 0,657; auxiliar: I = -0,113, p-valor = 0,537; técnico: I = 0,051, p-valor = 0,476; enfermero: I = -0,011, p-valor = 0,820).

**Figura 2-:** Mapa de incidencia de COVID-19 en profesionales de Enfermería en los estados brasileños. Brasil, 2020-2022

*A = Incidencia general por COVID-19; ^†^B = Incidencia por COVID-19 en Auxiliares de Enfermería; ^‡^C = Incidencia por COVID-19 en Técnicos de Enfermería; ^§^D = Incidencia por COVID-19 en Enfermeros

A pesar de haberse observado más casos en el año 2020, la COVID-19 en 2021 fue más letal para los profesionales de Enfermería (Tabla 1). La Figura 3 presenta la letalidad por año de notificación (2020 y 2021). En cuanto a la autocorrelación espacial, se observó para el año 2020: I = 0,241, p-valor < 0,001, y para el año 2021: I = 0,014, p-valor = 0,668. Estos resultados confirman la presencia de autocorrelación espacial global en el año 2020 y su ausencia en 2021. Esto indica que las UFs vecinas presentaron letalidad por COVID-19 similar; además, las tasas de letalidad fueron relativamente bajas en 2020, variando de 0,270 a 10,9 (Figura 3 A), encontrándose valores más altos en UFs de las regiones Norte y Centro-Oeste, con especial destaque para el estado de Amazonas, mientras que valores más bajos se observaron en los estados de Sudeste y Nordeste. Con el avance de la pandemia en 2021, se perdió el patrón espacial y hubo un aumento en la variabilidad de las tasas de letalidad, oscilando desde 0,0 para los estados de Piauí y Rio Grande del Norte hasta 76,62 para Amazonas.

En la Figura 4 se presentan mapas de la letalidad en profesionales de Enfermería. La Figura 4 A muestra la letalidad agrupada para todas las categorías, destacándose el estado de Amazonas, que presenta la mayor letalidad, de 28,74%, y el segundo mayor para el estado de Maranhão, con 6,7%. En todos los mapas de la Figura 4, el estado de Amazonas se destaca. Para auxiliares de Enfermería, la letalidad observada fue de 77,78%; para técnicos de Enfermería, fue de 38,57%; y para enfermeros, fue de 28,04%.

**Figura 3-:** Letalidad por COVID-19 de la Enfermería según año de notificación en los estados brasileños. Brasil, 2020-2021

*A = Letalidad por COVID-19 en 2020; †B = Letalidad por COVID-19 en 2021; No se confeccionó mapa para el año 2022, ya que hubo solo un fallecimiento durante el análisis

**Figura 4-:** Letalidad por COVID-19 en la Enfermería y según la categoría profesional en los estados brasileños. Brasil, 2020-2022

*A = Letalidad general por COVID-19; ^†^B = Letalidad por COVID-19 en Auxiliares de Enfermería; ^‡^C = Letalidad por COVID-19 en Técnicos de Enfermería; ^§^D = Letalidad por COVID-19 en Enfermeros

En cuanto a la letalidad entre los profesionales de Enfermería (Figura 4), los resultados para el Índice de Moran Global fueron: letalidad general: I = 0,084, p-valor = 0,086; Auxiliar: I = -0,133, p-valor = 0,349; Técnico: I = 0,097, p-valor = 0,037; Enfermero: I = 0,037, p-valor = 0,207. Estos resultados confirman la ausencia de correlación espacial global para la letalidad general, para los auxiliares de Enfermería y para los enfermeros, y la presencia de autocorrelación espacial para los técnicos. Entre los técnicos de Enfermería, la letalidad se destaca en el estado de Amazonas, con 28,04%, mientras que para los demás estados se observaron valores menores a 6%, registrándose los valores más bajos en estados costeros, desde Rio Grande do Sul hasta Sergipe.

## Discusión

Con el objetivo de profundizar en la comprensión sobre la distribución geográfica de la pandemia, el presente estudio mapeó los casos y fallecimientos por COVID-19 en profesionales de Enfermería, estimó sus tasas de incidencia y letalidad por región y unidades federativas, así como analizó la existencia de patrones espaciales entre las diferentes unidades de la federación.

A partir del análisis de la distribución espacial de casos y fallecimientos por COVID-19, es posible identificar áreas de mayor vulnerabilidad, relacionadas con la infraestructura de salud, el acceso a recursos y las condiciones de trabajo. Además, este análisis puede proporcionar elementos para la formulación de políticas públicas más eficaces y ajustadas a las necesidades locales. Al mapear estas desigualdades, es posible dirigir acciones que minimicen los impactos de la pandemia y preparen mejor el sistema de salud para futuras crisis.

La pandemia de COVID-19 representó un gran desafío para los profesionales de la salud, especialmente para los de Enfermería, quienes fueron esenciales en el enfrentamiento de la crisis. Constantemente expuestos al contacto con pacientes infectados e involucrados en procedimientos de riesgo, estos profesionales se volvieron altamente vulnerables a la enfermedad^(38-39)^.

La Enfermería, considerada una profesión históricamente femenina (85,2%), ha venido presentando una nueva tendencia hacia la masculinización, con aumento del contingente masculino^(40)^. Según los datos relacionados con el sexo, aunque las mujeres representan el doble de los fallecimientos en la Enfermería brasileña en números absolutos, los hombres enfrentaron un mayor riesgo de mortalidad por COVID-19, con una probabilidad de supervivencia un 45% menor y mayor riesgo de fallecimiento^(41)^. Además, se observa un mayor número de hospitalizaciones por COVID-19 entre el público masculino (54,4%), posiblemente debido a la negligencia en relación con la cuarentena, déficit en el autocuidado, propensión a enfermedades crónicas y, en consecuencia, mayor vulnerabilidad a la infección y a la gravedad de la enfermedad^(42-45)^.

Aunque la asociación entre edad avanzada y mayor riesgo de mortalidad por COVID-19 está ampliamente documentada, otros factores también desempeñan roles importantes^(38,43,46)^. Entre los profesionales de Enfermería, el rango etario de 31 a 40 años fue el más afectado por COVID-19 (41,2%), mientras que el 31% de los fallecimientos ocurrieron entre profesionales de 41 a 50 años, y el 28% en el rango de 51 a 60 años. Las tasas de letalidad aumentan con la edad. En los estados de Amazonas y Pará, las tasas de letalidad fueron superiores al 50% entre los profesionales de Enfermería en los rangos etarios más avanzados. La letalidad en el rango etario superior a 60 años, aunque la ocurrencia de fallecimientos es menor, resulta preocupante, pues refleja la pérdida de profesionales experimentados y en posiciones de liderazgo^(30,39,43)^.

En 2020, los casos y fallecimientos por COVID-19 fueron más numerosos, pero las tasas de letalidad en 2021 superaron las del año anterior en todas las regiones del país. Estados como Amazonas, Mato Grosso do Sul, Pará y Amapá presentaron letalidades particularmente elevadas, destacando la vulnerabilidad de la región Norte. El análisis a lo largo de los tres años de la pandemia, reveló una caída significativa en los casos y fallecimientos entre los profesionales de Enfermería, reflejando el impacto positivo de la vacunación iniciada en enero de 2021, ya que la inmunización demostró un efecto protector entre los profesionales de la salud^(47)^. Sin embargo, aunque la variable vacunados estaba disponible en la plataforma Observatorio de Enfermería, su registro inadecuado impidió su utilización en este estudio.

La evolución de los patrones de letalidad, según lo identificado por el Índice de Moran Global, reveló un cambio en la autocorrelación espacial entre 2020 y 2021. En 2020, la presencia de una autocorrelación espacial significativa indicaba *clusters* de alta letalidad en estados vecinos, lo que sugiere una respuesta más uniforme y coordinada a la emergencia sanitaria, con regiones próximas compartiendo condiciones similares de hospitalización y tratamiento. No obstante, en 2021, dicha correlación espacial se disipó, con la letalidad variando de forma más amplia entre las UFs. Esto sugiere que las respuestas regionales al COVID-19 se volvieron más heterogéneas, destacando la necesidad de estrategias de salud pública más adaptativas y sensibles a las condiciones locales.

Así, al establecer un análisis del escenario brasileño se deben observar las peculiaridades de cada una de sus macrorregiones. Durante la pandemia, las desigualdades regionales en Brasil se agravaron, y estos resultados podrían haber sido mitigados por una respuesta gubernamental adaptada a las realidades locales. Es importante considerar que, en Brasil, más de 7 millones de habitantes se encuentran a más de cuatro horas de un municipio polo de atención de alta complejidad, con equipos, camas de unidad de cuidados intensivos (UCI) disponibles y profesionales especializados. Estados como Amazonas, Pará y Acre presentan más del 20% de su población necesitando desplazarse por más de cuatro horas para recibir atención de alta complejidad^(20,48)^.

Otro dato relevante se refiere al número de beneficiarios de planes de salud. Según la Agencia Nacional de Salud Suplementaria (ANS), la región Sudeste es la que presenta el mayor número de beneficiarios, seguida por las regiones Sur, Nordeste y Centro-Oeste, siendo la región Norte la que presenta el menor número de vínculos con planes de salud^(49)^.

Además, la disponibilidad de camas por habitante varía significativamente entre las regiones. El Sudeste y el Centro-Oeste presentaron la mayor oferta de camas, con 11,50 y 10,07 camas por 100.000 habitantes, respectivamente, mientras que el Norte tuvo la menor cobertura, con 7,46 camas por 100.000 habitantes. La región Sur, por su parte, se destacó con la mayor tasa de camas exclusivas para COVID-19 en el SUS, con 5,18 camas por 100.000 habitantes^(50)^.

En medio del caos de la COVID-19, el acceso desigual a la salud pública y suplementaria pudo haber impactado significativamente los desenlaces de la pandemia. Regiones con mayor penetración de la salud pública y privada, como el Sudeste y el Sur, presentaron menores tasas de mortalidad y mayores porcentajes de cura, además de una mayor disponibilidad de camas de hospitalización. En contraposición, el Norte, región con menor disponibilidad, enfrentó mayores desafíos en el acceso a los servicios de salud, tratamiento y soporte clínico, lo que resultó en desenlaces más fatales^(49-50)^.

Brasil es un país de gran diversidad, con regiones como el Norte y el Nordeste presentando mayor vulnerabilidad socioeconómica. Aunque el Nordeste cuenta con atractivos turísticos y cierta presencia industrial, enfrenta desafíos significativos, como bajos indicadores sociales y un PIB reducido. La región posee la segunda mayor fuerza laboral en Enfermería, pero la cobertura de profesionales por habitante es inferior a la recomendada, similar a la del Norte^(31,36,51)^. En contraste, el Centro-Oeste, a pesar de ser menos industrializado y tener baja densidad poblacional^(31,51)^, el Distrito Federal presentó la mayor cobertura de enfermeros: 51,8 por 10.000 habitantes en 2020, lo que puede reflejarse en la calidad de los servicios de salud disponibles^(36)^. Un estudio sobre la distribución espacial de la COVID-19 en Brasil reveló que las desigualdades regionales influyeron en la diseminación y los impactos de la enfermedad, especialmente en áreas con menor disponibilidad de profesionales de la salud^(9)^.

La pandemia evidenció la fragilidad del acceso a la salud en la región Norte, que ya enfrentaba desafíos como bajos indicadores de desarrollo humano, grandes distancias y una infraestructura de transporte precaria^(52)^. A pesar de la menor cantidad de profesionales y de la baja densidad poblacional, la región Norte presentó las mayores tasas de letalidad. En comparación, el Sur, con altos indicadores sociales y aproximadamente el doble de camas de UCI por habitante, también contaba con más ventiladores mecánicos y médicos de UCI per cápita. Estos recursos son de difícil acceso y requieren años de capacitación especializada para la cualificación necesaria^(49,51,53)^.

La sobrecarga de los servicios de salud en Manaus, derivada de la concentración de la atención de alta complejidad en la capital, resultó en la ocupación total de las camas y en la escasez de insumos, impactando las estadísticas de COVID-19 en otros municipios y comprometiendo la capacidad de respuesta de los gestores. Con más del 50% de los pacientes hospitalizados originarios de otras localidades, la demanda de atención superó las fronteras municipales, forzando desplazamientos en busca de servicios^(48,52)^.

A pesar de que la región Norte presenta una población dispersa y menos centros urbanos, Manaus, con su alta densidad poblacional, enfrentó una demanda que superó la capacidad de recursos y profesionales, incluso con el apoyo de otros estados^(51)^. Estados de la Amazonía Legal, como Amapá, Amazonas y Roraima, registraron altas tasas de COVID-19, mientras que los recursos de salud, como ventiladores y camas de UCI, se asignaron de manera ineficiente, concentrándose en las capitales y exacerbando la desigualdad en el acceso a la salud en la región^(23)^.

El Amazonas, estado de mayor extensión territorial del país^(32)^, a pesar de presentar una distribución relativamente baja de casos, registró el segundo mayor número de fallecimientos en el país, quedándose detrás únicamente de São Paulo. Este escenario refleja las desigualdades regionales, como la escasez de profesionales de la salud, la ineficacia de las acciones gubernamentales y las desigualdades socioeconómicas. La concentración de los servicios de salud en las capitales, sumada a las precariedades existentes, aumenta los riesgos para los pacientes graves, especialmente debido a los desafíos de movilidad y a la distribución de insumos. La falta de conocimiento sobre medidas sanitarias y la baja adherencia a los protocolos agravan aún más la vulnerabilidad de la población, sobrecargando los servicios de salud^(52-53)^.

El análisis espacial de los casos y fallecimientos de COVID-19 en la Enfermería fue esencial para identificar disparidades geográficas y comprender la complejidad de la pandemia. Este análisis evidenció la necesidad de un enfoque multifacético en el planeamiento y la formulación de estrategias, especialmente en regiones vulnerables. Dicho enfoque es fundamental para optimizar las respuestas en salud pública, controlar la diseminación del virus y reducir de manera eficaz el impacto de la enfermedad^(53)^.

Los resultados del presente estudio aportan contribuciones significativas para el avance del conocimiento científico, especialmente por el uso de bases de datos específicas del área de la Enfermería, como el Observatorio de Enfermería y Enfermería en Números, que son poco exploradas en la literatura académica. A partir de estas fuentes, el estudio analizó la distribución geográfica de casos y fallecimientos por COVID-19 entre profesionales de Enfermería, incorporando datos sobre incidencia y letalidad por UF, identificando patrones espaciales a partir del índice de Moran Global y mediante la representación de mapas coropléticos, lo que permitió un análisis más profundo de los datos. Los hallazgos del estudio ofrecen nuevas perspectivas para futuras investigaciones, orientadas a las necesidades regionales de estos profesionales y al perfeccionamiento de las políticas públicas de salud.

Este estudio presenta algunas limitaciones, entre las que se encuentra la ausencia de variables de extrema relevancia en la base de datos, como datos socioeconómicos, raza/etnia y comorbilidades, así como el registro inadecuado en las fichas de notificación en variables como edad, categoría profesional y profesionales vacunados. Además, es necesario considerar el riesgo de subnotificación de casos y fallecimientos, un gran desafío en el análisis de los datos de COVID-19 en la Enfermería. Asimismo, el estudio presenta limitaciones inherentes a su diseño ecológico, especialmente en lo que se refiere a la posibilidad de falacia ecológica o sesgo de agregación, lo que significa que sus conclusiones son aplicables a agregados poblacionales y no pueden ser extrapoladas a nivel individual.

## Conclusión

La pandemia de COVID-19 evidenció las profundas desigualdades en el sistema de salud brasileño, destacando la precarización de las condiciones de trabajo de los profesionales de Enfermería y la distribución desigual de recursos. Regiones con infraestructura precaria y mayor vulnerabilidad socioeconómica enfrentaron desafíos más severos y resultados más adversos, en contraste con áreas más desarrolladas que presentaron desenlaces más favorables, debido a mejores indicadores sociales y a un acceso más amplio a la salud pública y suplementaria.

Aunque el análisis de los impactos de la pandemia mostró una reducción significativa de casos y fallecimientos, asociada al avance de la vacunación, la evaluación del efecto en los profesionales de Enfermería se vio comprometida por la falta de datos sobre la cobertura vacunal, debido al registro inadecuado en las fichas de notificación. Esto resalta la necesidad urgente de mejorar la recolección y el análisis de datos, además de fortalecer políticas públicas basadas en evidencias, con el objetivo de garantizar una protección más eficaz tanto para los profesionales de la salud como para la población en general.

El estudio también subraya la importancia de un enfoque adaptado a las realidades locales y de una respuesta gubernamental eficaz en la formulación de propuestas y estrategias orientadas a la promoción, protección y asistencia a la salud de los profesionales en la primera línea. Para combatir las desigualdades, es necesario reubicar recursos para fortalecer los servicios y garantizar la implementación de medidas preventivas eficaces. Este estudio busca fomentar la reflexión sobre cómo las disparidades regionales pueden impactar la pandemia de manera diversa, evidenciando la urgencia de un enfoque diferenciado y de una mayor inversión gubernamental en áreas socioeconómicamente más vulnerables.

## Data Availability

Todos los datos generados o analizados durante este estudio están incluidos en este artículo publicado.
